# Oncogenic KRAS mutations enhance amino acid uptake by colorectal cancer cells via the hippo signaling effector YAP1

**DOI:** 10.1002/1878-0261.12999

**Published:** 2021-06-18

**Authors:** Palanivel Kandasamy, Inti Zlobec, Damian T. Nydegger, Jonai Pujol‐Giménez, Rajesh Bhardwaj, Senji Shirasawa, Toshiyuki Tsunoda, Matthias A. Hediger

**Affiliations:** ^1^ Membrane Transport Discovery Lab Department of Nephrology and Hypertension, Inselspital University of Bern Switzerland; ^2^ Department of Biomedical Research University of Bern Switzerland; ^3^ Translational Research Unit (TRU) Institute of Pathology University of Bern Switzerland; ^4^ Department of Cell Biology Faculty of Medicine Fukuoka University Japan

**Keywords:** amino acid transporters, oncogene, SLC1A5/ASCT2, SLC38A2/SNAT2, SLC7A5/LAT1, solute carriers

## Abstract

Oncogenic KRAS mutations develop unique metabolic dependencies on nutrients to support tumor metabolism and cell proliferation. In particular, KRAS mutant cancer cells exploit amino acids (AAs) such as glutamine and leucine, to accelerate energy metabolism, redox balance through glutathione synthesis and macromolecule biosynthesis. However, the identities of the amino acid transporters (AATs) that are prominently upregulated in KRAS mutant cancer cells, and the mechanism regulating their expression have not yet been systematically investigated. Here, we report that the majority of the KRAS mutant colorectal cancer (CRC) cells upregulate selected AATs (SLC7A5/LAT1, SLC38A2/SNAT2, and SLC1A5/ASCT2), which correlates with enhanced uptake of AAs such as glutamine and leucine. Consistently, knockdown of oncogenic KRAS downregulated the expression of AATs, thereby decreasing the levels of amino acids taken up by CRC cells. Moreover, overexpression of mutant KRAS upregulated the expression of AATs (SLC7A5/LAT1, SLC38A2/SNAT2, and SLC1A5/ASCT2) in KRAS wild‐type CRC cells and mouse embryonic fibroblasts. In addition, we show that the YAP1 (Yes‐associated protein 1) transcriptional coactivator accounts for increased expression of AATs and mTOR activation in KRAS mutant CRC cells. Specific knockdown of AATs by shRNAs or pharmacological blockage of AATs effectively inhibited AA uptake, mTOR activation, and cell proliferation. Collectively, we conclude that oncogenic KRAS mutations enhance the expression of AATs via the hippo effector YAP1, leading to mTOR activation and CRC cell proliferation.

## Graphical abstract

Here, we demonstrate that oncogenic KRAS mutations upregulate the expression of selected amino acid transporters in colorectal cancer (CRC) cells through the hippo signaling effector YAP1. Upregulation of SLC1A5/ASCT2 and SLC38A2/SNAT2 led to intracellular l‐glutamine accumulation. l‐glutamine was reciprocally exported via SLC7A5/LAT1 in exchange for l‐leucine, a key activator of mTORC1. Intracellular l‐leucine led to the activation of mTORC1, thereby promoting KRAS mutant CRC cell proliferation.

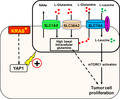

**Abbreviations**
AA: amino acid
AAT: amino acid transporter
ASCT2: alanine serine cysteine transporter 2
CRC: colorectal cancer
EAA: essential amino acids
GSEA: gene set enrichment analysis
GSH: glutathione
KRAS: kirsten rat sarcoma
LAT1: large neutral amino acid transporter 1
MCT: monocarboxylate transporters
MEF: mouse embryonic fibroblasts
mTOR: mammalian target of rapamycin
SLC: solute carrier
SNAT: sodium‐coupled neutral amino acid transporters
SOCS5/6: suppressor of cytokine signaling 5/6
TEAD: transcriptional enhanced associate domain transcription factors
YAP1: yes‐associated protein 1

## 1 Introduction

Tumor cells take up high amounts of amino acids (AAs) from the extracellular milieu in order to sustain proliferation and metastasis. Therefore, tumor cells upregulate certain AATs in order to facilitate the transport of AAs into and out of cells. AAs taken up by cancer cells are critical for the intra/extra cellular communication, biosynthesis of proteins, nucleotides, lipids, glutathione (GSH), glucosamine, and polyamines, as well as energy production and anaplerotic metabolism to replenish tricarboxylic acid (TCA) cycle intermediates [1]. Recent reports revealed that cancer cells exhibit alterations in expression and/or function of specific AATs in order to take up exogenous AAs depending on the specific requirement of the tumor subtypes [1, 2]. One of the well‐known characteristics of tumor cells is accumulation of glutamine, leading to glutamine addiction [3, 4, 5]. Notably, glutamine is a conditionally essential amino acid (EAA) that contributes even at relatively low concentrations toward proliferation of normal healthy cells. However, tumor cells require much higher levels of glutamine to survive [6].

To acquire AAs from the extracellular milieu, mammalian cells expresses a variety of AATs. These are grouped into SoLute Carrier (SLC) families based on sequence identity and other criteria [7]. To date, over 65 different SLC families have been identified that display a large phylogenetic diversity [7]. Of these, 11 families contain AATs, giving rise to at least 66 AATs [1]. These are mostly expressed in a tissue‐specific manner, controlling particular cellular functions [8]. Quiescent cells express AATs at basal levels because they only require low concentrations of AAs, whereas tumor cells require high amounts of AAs and therefore upregulate specific sets of AATs for increased uptake of the required AAs [9]. Notably, not all tumor subtypes upregulate the same sets of AATs, as each subtype has specific AA requirements and thus upregulates unique sets of AATs [10]. For instance, SLC6A14 is specifically overexpressed in estrogen receptor‐positive breast cancer cells [11, 12]. To date, there are two different AATs (SLC1A5/ASCT2 and SLC7A5/LAT1) that have been reported to play important roles in the progression of different types of human cancers through mTOR activation [13, 14]. However, the association of these AATs with different molecular/histological subtypes of cancers is largely unknown. Both, SLC7A5/LAT1 and SLC1A5/ASCT2 play key roles in the upstream of the mTOR pathway in maintaining AA homeostasis in response to changes in intra‐ and extracellular AAs levels. In an earlier study, it has been reported that the cellular uptake of glutamine through SLC1A5/ASCT2 is the rate‐limiting step for uptake and maintenance of optimal intracellular concentrations of EAAs, which also involves SLC7A5/LAT1 for activation of mTOR [5]. However, recent reports suggest that the rate‐limiting role of SLC1A5/ASCT2 in glutamine uptake is difficult to explain because SLC1A5/ASCT2 is an exchanger and thus net accumulation of glutamine requires some other AATs [15, 16]. Furthermore, it has been demonstrated that SLC38A1/SNAT1 and SLC38A2/SNAT2 activity is essential to mediate net glutamine uptake and glutaminolysis in cancer cells, whereas SLC1A5/ASCT2 and SLC7A5/LAT1 coordinate intracellular amino acid pools [17]. Altogether, previous reports suggests that cancer cells expresses different sets of AATs and there may be cancer subtype‐specific expression patterns of AATs. Hyperactivation of mTOR by amino acids in cancer cells is one of the hallmarks of the tumor progression, leading to rapid proliferation of cancer cells. Oncogenic KRAS signaling has been reported to affect glutamine transport, energy metabolism, and cancer cell proliferation through mTOR activation [18, 19].

KRAS is one of the most widely mutated oncogene in human colorectal cancers (CRCs). KRAS encodes a GTPase that transmits signals from growth factor receptors to downstream signaling cascades. Mutations in KRAS enhance GTP binding, resulting in a constitutively active form of KRAS, sufficient for oncogenic transformation and induction of cancer development. A major mechanism underlying KRAS‐driven oncogenic transformation is the constitutive stimulation of the pro‐proliferative RAF‐MEK‐ERK pathway and the pleiotropic PI3K‐AKT pathway to prevent apoptosis. Ultimately, inhibition of MEK‐ERK pathways has been considered for the treatment of KRAS mutant CRCs. However, KRAS mutant cancer cells develop resistance against therapeutic agents through altered metabolic pathways. Notably, KRAS mutant cancer cells have been shown to develop anticancer drug resistance through hyperactivation of mTOR signaling [20]. These reports highlight the need for identification of specific AATs that support the mTOR activation in KRAS mutant CRCs. Several previous reports have shown that targeting KRAS mutant cancers relies on the identification of unique targets [21, 22, 23]. Interestingly, oncogenic KRAS mutations were shown to increase glucose uptake in cancer cells and lactate production through upregulation of glycolytic genes encoding glucose transporter SLC2A1/GLUT1 and the monocarboxylate transporters SLC16A1/MCT1, SLC16A7/MCT2, and SLC16A3/MCT4 [24, 25, 26]. Interestingly, a previous report showed upregulation of SLC1A5/ASCT2 in KRAS mutant CRC patients [27].

Altogether, it is apparent that AATs play a critical role in fulfilling the specific AA requirements in cancer cells, whereas the impact of oncogenic KRAS mutations on the expression and function of specific AATs in CRC cells are largely unknown. Therefore, we have undertaken an in‐depth analysis in order to investigate the link between the expression and function of AATs and the oncogenic KRAS mutation status of CRCs.

In addition, previous studies have shown that the transcription factors (TFs), c‐MYC, ATF4, and E2F, regulate the expression of AATs such as SLC1A5/ASCT2 or SLC38A5/SNAT5, and affect mTOR activation [28]. Interestingly, a recent report indicates that the activity of these TFs (MYC, E2F, and TEAD) are linked to hippo‐pathway effector YAP1 [29]. YAP1 is a transcriptional coactivator and a component of the hippo tumor suppressor pathway, required for the transcription of genes involved in cell proliferation, metabolism, tissue homeostasis, and organ size. YAP1 has recently been shown to play a distinct role in oncogenic KRAS mutant cells [30], and it has been reported that oncogenic KRAS is involved in the stabilization of YAP1 [30, 31, 32]. Notably, recent studies also reported that upregulation of YAP1 is involved in cancer metabolism and mTOR activation by altering specific genes involved in metabolic pathways [33, 34].

The hippo tumor suppressor pathway includes the MST1/2‐LATS1/2 kinases cascade, which phosphorylates YAP1 and segregates YAP1 in the cytosol for degradation [35]. This pathway is a key barrier to RAS‐mediated oncogenic transformation. The hippo pathway triggers YAP1 degradation through the βTrCP‐SCF ubiquitin ligase complex. On the other hand, the oncogenic RAS acts oppositely, to promote YAP1 stability through downregulation of the ubiquitin ligase complex substrate recognition factors SOCS5/6 [30], leading to nuclear translocation of YAP1. Following nuclear translocation, YAP1 binds to its nuclear partners, in particular TFs such as TEAD, thereby promoting cell proliferation by inducing the expression of genes that affect cell survival and metabolism.

Thus, given the emerging oncogenic roles of YAP1 in cancer metabolism and KRAS mutant tumors, we also aimed to investigate the role of YAP1 in the regulation of AATs expression and function in KRAS mutant CRC cells.

## 2 Materials and methods

### 2.1 Cell lines and reagents

Unless otherwise stated, chemicals were obtained from Sigma‐Aldrich (St. Louis, MO, USA), and cell culture reagents and cell growth medium from Gibco (Grand Island, NY, USA). Colorectal cancer cell lines (SW480, SW620, DLD‐1, HCT116, LS174T, T84, LOVO, Caco‐2, and Colo320) were from ATCC. DKO‐4, HKe3‐Parental (KRAS^WT/G13D−^), HKe3‐KRAS wt (KRAS^WT/WT+^), and HKe3‐KRAS mutant (KRAS^WT/G13D+^) cell lines were provided by Senji Shirasawa [23, 36, 37]. Mycoplasma contamination tests were performed regularly, and cells with low passage numbers were used for the experiments. All CRC cell lines were maintained at 37 °C in humidified 5% CO_2_ in air, and stock cells were maintained in RPMI‐1640 medium (Cat. No: 21875) containing 2 mm l‐glutamine, supplemented with 10% FBS (Cat. No: 10270106; Gibco, Life Technologies).

### 2.2 Plasmids and shRNA cloning

All shRNA primers were synthesized by Microsynth AG, Balgach, Switzerland. shRNA primers (Table S1) were cloned into pSuperior‐neo (Cat. No: VEC‐IND‐0004; Oligoengine, Seattle, WA, USA) according to manufacturer's instructions. Sanger sequencing (Microsynth AG) was used to verify the correct cloning of shRNA into the vectors.

### 2.3 shRNA transfection for the knockdown of SLCs and KRAS

SW480, SW620, DLD‐1, and HCT116 cell lines were transfected with regulatory vector that expresses the tetracycline repressor (TetR), pcDNA™6/TR (Cat. No: V1025‐20; Invitrogen, Life Technologies, Carlsbad, CA, USA), and TetR expressing cells were generated by blasticidine S antibiotic selection. Then, the cells were transfected with the pSuperior‐neo vector containing the specific shRNAs (Table S1) to knock down the amino acid transporters and KRAS. All plasmid transfections were carried out using lipofectamine 2000 (Invitrogen, Life Technologies). Unless otherwise stated, all experiments with shRNA‐expressing cell lines were performed using RPMI‐1640 medium containing 10% of Tet‐system approved FBS (Cat.no: 631106; Clontech, Takara, Mountain View, CA, USA) and 2 mm glutamine.

### 2.4 Isogenic mutant KRAS‐expressing mouse embryonic fibroblasts (MEF‐KRAS)

Mouse embryonic fibroblasts stably expressing isogenic wt KRAS and mutant KRAS were a kind gift from the National Cancer Institute (NCI), NIH, USA, as part of the Ras initiative. MEF‐KRAS cell lines were maintained in DMEM with high glucose (Cat. No: 41965), supplemented with sodium pyruvate (Cat. No: S8636; Sigma‐Aldrich) and 10% FBS.

### 2.5 XTT cell proliferation assay

One thousand cells per well were seeded in 96‐well plates, incubated overnight, and different treatments were applied (doxycycline‐induced shRNA and nutrient deprivation). The cell proliferation was measured after the treatment protocols by using the XTT colorimetric assay (Cat. No: X4626; Sigma‐Aldrich). The absorbance value of each well was measured by a microplate reader at 450–650 nm. Each experiment was repeated three times.

### 2.6 Crystal violet clonogenic assay

To assess the colony forming efficiency of CRC cells under different concentrations of L‐glutamine and in response to specific knockdowns of KRAS and AATs, 300’ cells/well were seeded into six well plates and the medium was refreshed every three days and maintained for up to two weeks. At the end of the procedure, the colonies were washed with PBS at least three times, stained with crystal violet and photographed. The colony numbers were counted using Fiji Image J (NIH, USA), a open source image processing software.

### 2.7 Amino acid uptake assays

Live‐cell amino acid uptake assays using cancer cell lines were carried out in white 96‐well plates (Corning, Costar, Kennebunk, ME, USA). 96‐well plates were coated with poly‐d‐lysine prior to the assay. Cells were plated at a density of 10 000 cells per well 24 h prior to carrying out the assay. Each set of conditions was replicated at least three times. Cells were washed three times with 100 µL of transport assay buffer (containing 137 mm NaCl, 5.1 mm KCl, 0.77 mm KH_2_PO_4_, 0.71 mm MgSO_4_·7H_2_O, 1.1 mm CaCl_2_, 10 mm d‐glucose, and 10 mm HEPES) to remove cell culture media. ^3^H‐amino acid (0.05 µCi) and 100 µL of nonradioactive amino acids in the same buffer was added to the cells and allowed to incubate for 20 min at 37 °C. Following the incubation period, ^3^H‐glutamine or ^3^H‐leucine‐containing buffer was removed and the cells were washed three times with assay buffer. For reading, 100 μL of scintillation cocktail (Microscint 20; Perkin Elmer, Groningen, The Netherlands) was added and the plates were counted on a scintillation counter (Topcount, Perkin Elmer).

### 2.8 siRNA knockdown of YAP1

YAP1 silencing was performed in CRC cells by transfecting ON‐TARGET plus siRNAs against YAP1 (Cat#J‐012200‐07, Ca#J‐012200‐08; Dharmacon, St. Louis, MO, USA) or control siRNA (AllStar negative Control siRNA; Dharmacon) using Lipofectamine 2000 (Invitrogen) according to the manufacturer's protocol.

### 2.9 Inhibitors and treatments

YAP1/TEAD1 interaction inhibitor, verteporfin (Cat. No: SML0534) was obtained from Sigma‐Aldrich. Pharmacological inhibitors of amino acid transporters, V‐9302 (Cat#. S8818) and JPH203 (Cat#. S8667) were obtained from SelleckChem, Houston, TX, USA. Stock solutions of Verteporfin, JPH203 and V‐9302 were prepared using DMSO and stored at −20 °C. MEF‐KRAS cells were treated with verteporfin at 2 µg·mL^−1^ for 48 h, and the total cell lysates were prepared using RIPA buffer for the western blot analysis.

### 2.10 RNA extraction, cDNA synthesis, and real‐time quantitative PCR

RNA extractions were performed using RNeasy kit (Qiagen, Hilden, Germany) according to the manufacturer's guidelines. RNA (1 μg) was used for each complementary DNA synthesis reaction (Cat. No: N8080234, TaqMan reverse transcription reagents; Applied Biosystems, Carlsbad, CA, USA). Quantitative real‐time PCRs were performed with 20 ng of cDNA, gene‐specific primers (Table S2) and SYBR Green PCR reagents (Applied Biosystems) using a viiA7 real‐time thermal cycler. The cycle threshold values of the target genes were normalized to cycle threshold values of GAPDH.

### 2.11 Antibodies and western blotting

After completing the treatment protocols, cells were washed three times with ice‐cold PBS and lysed in ice‐cold RIPA buffer [1% sodium deoxycholate, 0.1% SDS, 1% Triton X‐100, 10 mm Tris (pH 8.0), and 150 mm NaCl] supplemented with protease inhibitor cocktail (Roche, Indianapolis, IN, USA). Equal amounts of protein (20–30 µg) were loaded on 8–12% SDS/PAGE gels and followed by transfer to PVDF membrane (Merck Millipore, Darmstadt, Germany). Immunoblotting was performed with the following antibodies: rabbit polyclonal anti‐SLC38A2 (Cat. no. HPA035180) antibody was purchased from Sigma‐Aldrich, USA. Mouse monoclonal antibodies against YAP (Cat. no. sc‐271134), pan‐Ras (Cat. no. sc‐166691), KRAS (Cat. no. sc‐30), and β‐Actin were purchased from Santa Cruz Biotechnology (Heidelberg, Germany). Rabbit polyclonal antibody against SLC1A5/ASCT2 (Cat. no. 5345), rabbit monoclonal antibodies against TEAD (Cat.no. 13295S), p‐MEK1/2 (Cat. no. S217/221), total‐MEK1/2 (Cat. no. 9122S), phospho‐S6 ribosomal protein (Ser235/236, Cat. no. 2211), and ribosomal S6 kinase (Cat. no. 2217S) were purchased from Cell Signaling Technology, Danvers, MA, USA. Polyclonal rabbit anti‐LAT1/SLC7A5 antibody was provided by TransGenic Inc. (Kumamoto, Japan).

### 2.12 Gene expression analysis and patient survival data using public datasets

Data on mRNA expression levels of AATs (SLC1A5, SLC7A5, and SLC38A2) in normal and COAD (colon adenocarcinoma) specimens were retrieved from the TCGA samples at the UALCAN database [38]. Correlation of mRNA expression levels of the AATs (SLC1A5, SLC7A5, and SLC38A2) with KRAS mutations in CRC patient samples were derived from the Gene expression Omnibus (NCBI‐GEO) public dataset (GSE39582). Colorectal cancer patients' overall survival probability data were generated based on the mean expression levels of the AATs (SLC1A5, SLC7A5, and SLC38A2) from the database, kmplot.com [39].

### 2.13 Gene set enrichment analysis

Microarray dataset from HCT116 cells transfected with non‐targeting siRNA and YAP1 siRNA (GSE92335), RNA‐seq dataset from Hke3‐KRAS^WT/WT+^ and Hke3‐KRAS ^WT/G13D+^ cell lines (GSE110649), and DLD‐1 cell line expressing homogeneous KRAS^G13D^ or KRAS^WT^ (GSE119197) were downloaded from the NCBI‐GEO database. Gene set enrichment analysis (GSEA) for major hallmark pathways and oncogenic signatures were performed using gsea_4.1.0 software available from the Broad Institute (http://www.broadinstitute.org/gsea/downloads.jsp) [40, 41]. Gene Set Enrichment Analysis between experimental groups was performed as previously described, using the Broad Institute software with default parameters, 1000 permutations of gene sets, and Signal2Noise metric for ranking of genes.

### 2.14 Statistical analysis

Three independent experiments were performed prior to statistical analysis. The data represent the mean ± SEM. *P < 0.05, **P < 0.01, and ***P < 0.001 by one‐ or two‐way ANOVA were considered statistically significant.

## 3 Results

### 3.1 KRAS mutant CRC cells exhibit increased AA uptake to support their proliferation

**Fig. 1:**
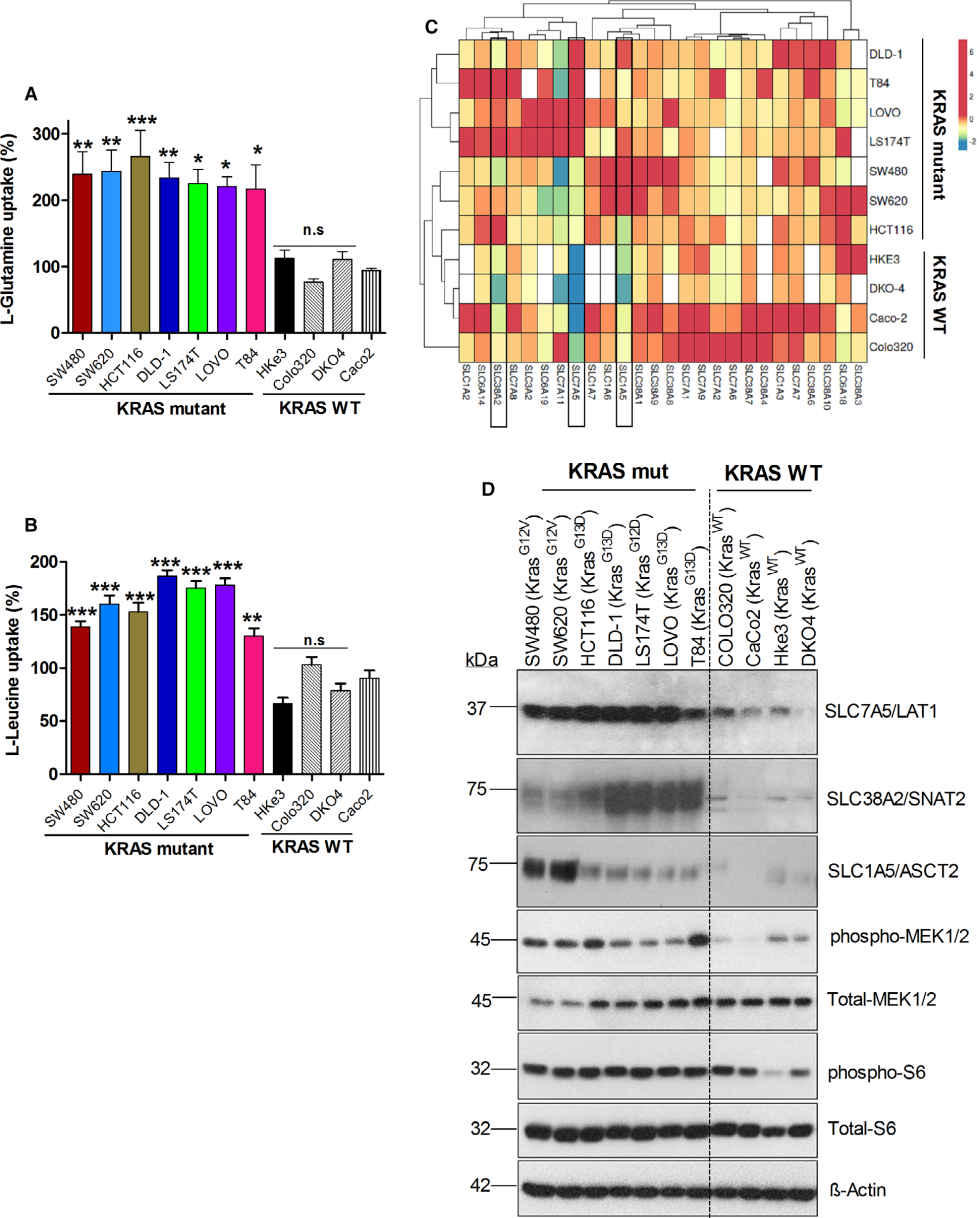
Glutamine and leucine uptake in KRAS mutant CRC cells associated with the expression of selected AATs: (A, B) l‐glutamine and l‐leucine uptake levels in KRAS mutant or wt CRC cell lines at 20 min, determined by radiolabeled amino acid transport assay. Data are presented as the mean ± SEM from three independent experiments (*n* = 3). Statistical comparisons were computed by one‐way ANOVA (*P < 0.05, **P < 0.01, ***P < 0.001). (C) Heat map shows mRNA expression levels (fold change) of SLC‐amino acid transporter genes in different CRC cell lines from three independent biological replicates (*n* = 3). (D) Western blot images show the protein expression of AATs (SLC38A2, SLC7A5, and SLC1A5), total‐MEK1/2, phospho‐MEK1/2, total ribosomal S6, and phospho‐ribosomal S6 protein in different CRC cell lines. β‐actin was used as protein loading control. All the experiments were performed three times independently, and the blots shown are representative of three independent replicates.

While cancer cells consume glutamine at exceedingly high rates to meet their energetic and biosynthetic requirements for proliferation [19], it is not clear yet whether oncogenic KRAS mutations influence glutamine transport. To clarify this, we measured l‐glutamine uptake in a panel of CRC cell lines with and without KRAS mutations, using a l‐[^3^H]‐glutamine radioisotope transport assay. The results reveal increased l‐glutamine uptake for KRAS mutant CRC cells, compared with the KRAS wt cells (Fig. 1A). To evaluate whether the observed increase in l‐glutamine uptake by KRAS mutant CRC cells also correlate with general increase in the uptake of EAAs, we measured l‐leucine uptake in the same CRC cell line panel. As expected, the KRAS mutant CRC cells showed an increase in l‐leucine uptake compared with KRAS wt cells (Fig. 1B). To examine whether the increased glutamine uptake is linked to increased proliferation of KRAS mutant CRC cells, cells were grown in medium containing different concentrations of glutamine (0.5–5 mm) for 5 days. The results show that the KRAS mutant CRC cell proliferation rate significantly decreased with reduced concentration of (0.5 mm) glutamine, whereas the KRAS wt CRC cell lines exhibit a less significant dependence on extracellular glutamine (Fig. S1A). These results suggest that KRAS mutant CRC cells have a higher demand for glutamine compared with the KRAS wt cells. In addition, a colony‐forming assay was performed on the same panel of CRC cells after they were cultured in medium containing different glutamine concentrations for two weeks (Fig. S1B). The results show that colony‐forming efficiency of all the KRAS mutant CRC cells was gradually decreased in a glutamine dose‐dependent manner. In contrast, the colony‐forming efficiency of KRAS wt cell lines exhibit a less significant glutamine dependence, even after culturing them in medium containing 0.5 and 0.2 mm glutamine. Overall, these data demonstrate that KRAS mutant cells have an enhanced demand for glutamine in order to support their proliferation.

### 3.2 Upregulation of selected AATs (SLC1A5/ASCT2, SLC7A5/LAT1, and SLC38A2/SNAT2) associated with oncogenic KRAS mutations in CRC cells

Given the significant dependence of KRAS mutant tumor cell growth on glutamine and leucine supply, it is reasonable to expect that these cells upregulate specific AATs to meet this requirement. We further assume that, because the oncogenic pathways responsible for the initiation and propagation of tumors vary markedly from one tumor subtype to another, KRAS mutant CRC cells upregulate specific sets of AATs to support growth and proliferation. Therefore, we analyzed the mRNA expression levels of all AAT genes by qPCR in our CRC cell line panel. As shown in Fig. 1C, three different AATs, SLC1A5/ASCT2, SLC7A5/LAT1, and SLC38A2/SNAT2, are upregulated at the mRNA level in many of the KRAS mutant CRC cell lines as compared to KRAS wt cell lines. To validate this finding, we determined the protein expression of these AATs by western blot analysis. Western blot analysis also showed significant increase in the protein expression levels of SLC1A5/ASCT2, SLC7A5/LAT1, and SLC38A2/SNAT2 in most of the KRAS mutant CRC cells (Fig. 1D). In addition, we show upregulation of these AATs in KRAS mutant cells to be associated with increased phosphorylation of MEK1/2, a downstream target of mutant KRAS and ribosomal protein S6, a downstream target of mTOR (Fig. 1D). These data indicate that upregulation of these three AATs (SLC1A5/ASCT2, SLC7A5/LAT1, and SLC38A2/SNAT2) likely contribute to enhanced cellular accumulation of glutamine and leucine in KRAS mutant CRC cells, followed by mTOR activation. In support of this, recent reports revealed that metabolic dependencies and changes in expression of metabolic genes are linked to the mutant KRAS oncogenes in cancer cells [24, 42, 43]. We therefore wanted to investigate whether oncogenic KRAS mutations influence expression/functions of these three AATs and their roles in the CRC cell proliferation.

### 3.3 Knockdown of oncogenic KRAS inhibits the expression and function of AATs and mTOR activation in CRC cells

**Fig. 2:**
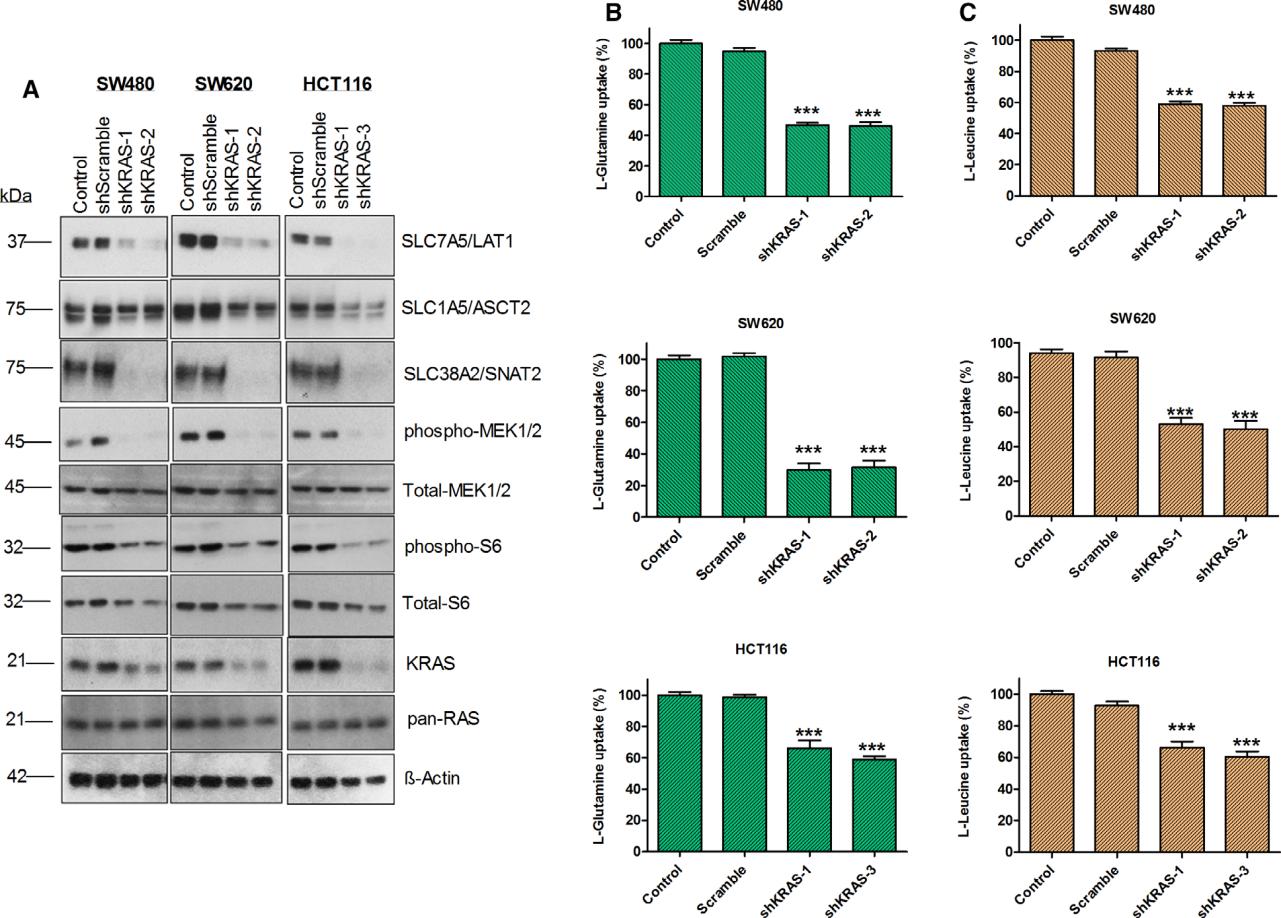
Knockdown of oncogenic KRAS inhibits the expression of AATs, AA uptake, and mTOR activation in CRC cells. (A) Western blot images show the impact of knockdown of oncogenic KRAS on the protein expression of AATs (SLC7A5/LAT1, SLC1A5/ASCT2, and SLC38A2/SNAT2), KRAS, pan‐RAS, total‐MEK1/2, phospho‐MEK1/2, total‐S6, and phospho‐S6 ribosomal protein in CRC cell lines, SW480, SW620, and HCT116. β‐actin was used as protein loading control. All the experiments were performed three times independently, and the blots shown are representative of three independent replicates. (B, C) Impact of knockdown of KRAS on the l‐glutamine and l‐leucine uptake, measured by radioisotope‐based membrane transport assays. Data are presented as the mean ± SEM from three independent experiments (*n* = 3). Statistical comparisons were computed by one‐way ANOVA (*P < 0.05, **P < 0.01, ***P < 0.001).

As already alluded to, tumor cells are highly dependent on macromolecular precursors including AAs, nucleic acids, and fatty acids to sustain growth [44]. Previous studies have reported that the corresponding biosynthetic metabolic pathways are frequently upregulated in tumors due to the gain‐of‐function mutations in the oncogenes and/or the loss‐of‐function mutations in the tumor suppressors [45, 46, 47]. As mentioned earlier, a recent report showed upregulation of SLC1A5/ASCT2 in KRAS mutant CRC patient samples [27]. However, there is no detailed study whether oncogenic KRAS mutations regulate the expression of other AATs in order to acquire increased levels of AAs. To evaluate whether oncogenic KRAS influence the expression of specific AATs, shRNAs targeting KRAS were transfected into KRAS mutant CRC cell lines (SW480, SW620, and HCT116) and determined the changes in the expression and functions of AATs. Our results show that the knockdown of oncogenic KRAS results in a significant decrease in the protein expression levels of three AATs (Fig. 2A). In addition, our data show that the knockdown of KRAS decreases the activation of ribosomal protein S6 and MEK1/2 (Fig. 2A). To further substantiate these results at the cellular level, we measured the uptake of l‐glutamine and l‐leucine following the shRNA‐mediated knockdown of KRAS in CRC cells. As expected, knockdown of oncogenic KRAS significantly inhibits the uptake of glutamine and leucine in CRC cells (Fig. 2B,C). Furthermore, to correlate this effect with the proliferation of CRC cells, we determined the proliferation of cells upon the knockdown of oncogenic KRAS. The data reveal a more significant change in proliferation rate upon the knockdown of KRAS (Fig. S2A).

Furthermore, to ensure whether the knockdown of KRAS in CRC cells constantly affects the cell proliferation, we cultured the cells for up to two weeks after the shRNA‐mediated knockdown and determined the colony‐forming efficiencies by crystal violet staining. Our data show that knockdowns of KRAS more dramatically inhibited the colony‐forming efficiency of CRC cells (Fig. S2B). Collectively, our data reveal that oncogenic KRAS alters the expression of AATs such as SLC1A5/ASCT2, SLC7A5/LAT1, and SLC38A2/SNAT2 and thereby increase the uptake of AAs to support cancer cell proliferation through mTOR activation.

### 3.4 Overexpression of mutant KRAS induces the expression and functions of AATs and mTOR activation in KRAS wt CRC cells and mouse embryonic fibroblasts

**Fig. 3:**
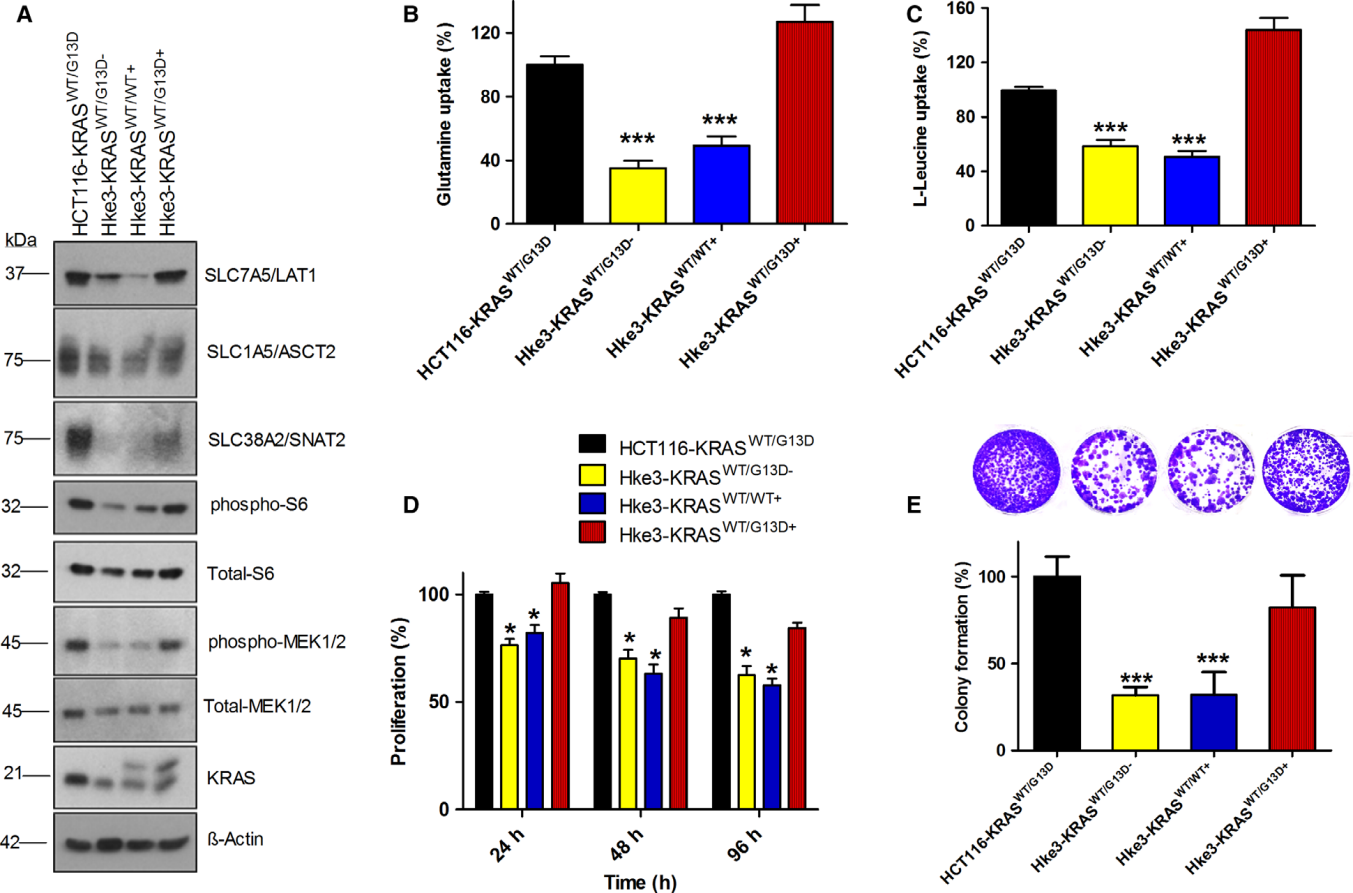
Overexpression of mutant KRAS induce the expression of AATs, AA uptake, and mTOR activation in KRAS wt cell line HKe3. (A) Western blot images show the expression of AATs (SLC7A5/LAT1, SLC1A5/ASCT2, and SLC38A2/SNAT2), phospho‐MEK1/2, phospho‐S6 ribosomal protein, and KRAS in HCT116‐KRAS^WT/G13D^, Hke3‐KRAS^WT/G13D−^, Hke3‐KRAS^WT/WT+^, and Hke3‐KRAS^WT/G13D+^ cells. KRAS blot show two bands in the lane 3 and 4, as the Hke3‐KRAS^WT/WT+^ and Hke3‐KRAS^WT^/^G13D+^ cells express HA‐tagged and untagged KRAS. All the experiments were performed three times independently, and the blots shown are representative of three independent replicates. (B, C) l‐glutamine and l‐leucine uptake levels in HCT116 and HKe3 cell lines expressing KRAS wt and KRAS mutation. (D) Proliferation rate of HCT116‐KRAS^WT/G13D^, Hke3‐KRAS^WT/G13D−^, Hke3‐KRAS^WT/WT+^, and Hke3‐KRAS^WT/G13D+^ cells at 24, 48 and 96 h. (E) Bar graph show colony‐forming efficiency of HCT116‐KRAS^WT/G13D^, Hke3‐KRAS^WT/G13D−^, Hke3‐KRAS^WT/WT+^, and Hke3‐KRAS^WT/G13D+^ cells. Data are presented as the mean ± SEM from three independent experiments (*n* = 3). Statistical comparisons were computed by one‐way ANOVA (*P < 0.05, **P < 0.01, ***P < 0.001).

To understand the specific effects of wt and mutant KRAS on the expression and functions of AATs, we used a human CRC cell line with mutant KRAS (HCT116‐KRAS^WT/G13D^) and its isogenic cell line (HKe3 parental‐KRAS^WT/G13D−^), the independent HCT116 clone in which the endogenous mutant KRAS^G13D^ allele was deleted through targeted homologous recombination [36]. In addition, we employed the HKe3 cell line in which either HA‐tagged KRAS wt (Hke3‐KRAS^WT/WT+^) or KRAS^G13D^ (Hke3‐KRAS^WT/G13D+^) is stably expressed. The Hke3‐KRAS^WT/WT+^ and Hke3‐KRAS^WT/G13D+^ cell lines have been extensively characterized previously [23, 37], and both cell lines also express a genome‐encoded copy of KRAS wt. Endogenous KRAS wt was expressed at similar levels in both Hke3‐KRAS^WT/WT+^ and Hke3‐KRAS^WT/G13D+^ cell lines. However, the mutant KRAS protein expression level of was higher in Hke3‐KRAS^WT/G13D+^ cells than the expression level of wt KRAS protein in Hke3‐KRAS^WT/WT+^ [23, 37]. Therefore, these paired cell lines basically only differ in the mutational status of the KRAS gene and, thus, this allowed us to investigate the specific effect of mutant‐ and wt‐KRAS on the expression and functions of AATs. Interestingly, our data show that the protein expression levels of AATs (SLC1A5/ASCT2, SLC7A5/LAT1, and SLC38A2/SNAT2), mTOR activation (Fig. 3A), and the uptake levels of l‐glutamine and l‐leucine (Fig. 3B,C) were significantly increased in KRAS mutant cell lines (HCT116‐KRAS^WT/G13D^ and HKe3‐KRAS^WT/G13D+^) as compared to KRAS wt cell lines (HKe3‐KRAS^WT/G13D−^ and Hke3‐KRAS^WT/WT+^). In addition, we show that the upregulation of AATs and increased uptake of AAs strongly associated with the increased phosphorylation of MEK1/2 and ribosomal protein S6 in KRAS mutant cell lines (HCT116‐KRAS^WT/G13D^ and HKe3‐KRAS^WT/G13D+^), as compared to KRAS wt cell lines (HKe3‐KRAS^WT/G13D−^ and Hke3‐KRAS^WT/WT+^). Furthermore, our data show that KRAS mutation induced AATs upregulation, AA uptake and mTOR activation significantly associated with cell proliferation and colony formation (Fig. 3D,E).

We then performed an additional validation experiments using MEFs expressing isogenic wt KRas or mutant KRas oncogenes individually. In these MEFs, endogenous HRas and NRas alleles are constitutively knocked out, whereas the Kras lox/lox alleles are under the control of a resident 4‐hydroxytamoxifen (4OHT)‐dependent recombinase. Then, the RAS‐less MEFs are transduced with different KRAS mutants, including the most common mutations detected in human CRCs (KRas^G12C^, KRas^G12D^, KRas^G12V^, and KRas^G13D^). Therefore, we utilized these model cell lines for validating our hypothesis, that is, to understand the specific effect of KRAS mutations without the interference of HRAS and NRAS. Interestingly, western blot analysis of AATs in these MEFs reveals that mutant KRAS‐expressing MEFs exhibit significant upregulation of AATs and activation of mTOR, compared with wt KRAS‐expressing MEFs (Fig. S3A). Furthermore, upregulation of AATs in MEFs upon mutant KRAS overexpression was accompanied by increased uptake of l‐glutamine and l‐leucine (Fig. S3B,C). To investigate further whether the induction of AATs expression in KRAS mutant MEFs is necessary for the increased uptake of AAs and sustained proliferation, we cultured MEFs in medium containing various concentrations of glutamine and measured the rate of proliferation by cell proliferation and colony formation assays. Our data reveal that MEF cells expressing KRAS mutant oncogenes are highly sensitive to glutamine removal, whereas KRAS wt expressing MEFs did not show significant changes in proliferation as well as colony formation rate (Fig. S3D–F). Overall, these data reveal that oncogenic KRAS mutations induce the expression of selected AATs and increase the uptake of AAs, in order to support cell proliferation through mTOR activation.

### 3.5 Knockdown of AATs (SLC1A5/ASCT2, SLC7A5/LAT1, and SLC38A2/SNAT2) inhibits the AA uptake, mTOR activation, and proliferation in CRC cells

**Fig. 4:**
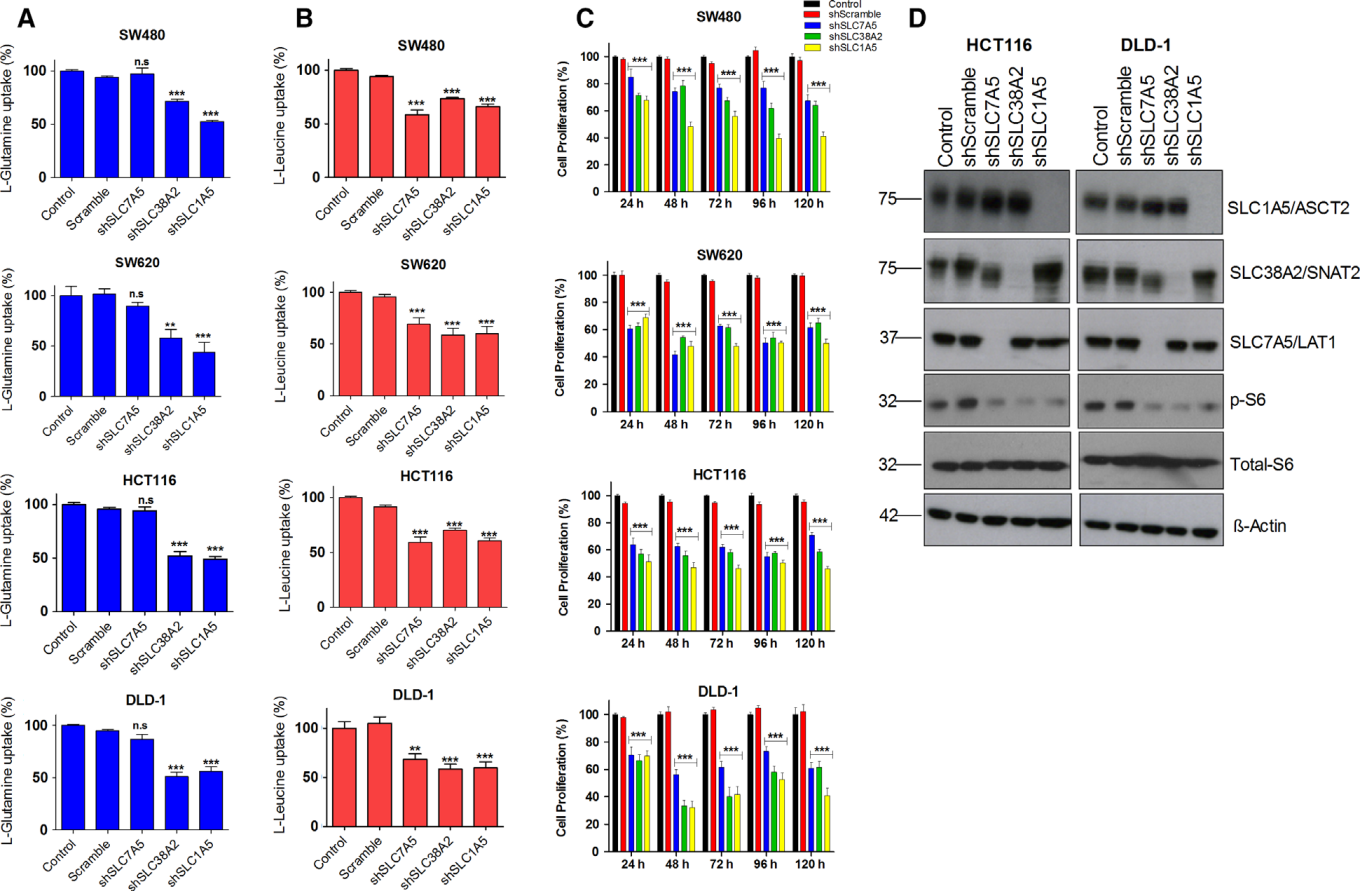
Knockdown of AATs inhibits AA uptake, mTOR activation, and proliferation in CRC cells: (A, B) Changes in the l‐glutamine and l‐leucine uptake levels in different CRC cell lines (SW480, SW620, HCT116, and DLD‐1) following the transfection of shRNAs against SLC7A5, SLC38A2, and SLC1A5. (C) Impact of knockdowns of SLC1A5, SLC7A5, and SLC38A2 genes on the proliferation of CRC cell lines SW480, SW620, HCT116, and DLD‐1 at 24, 48, 72, 96 and 120h. Data are presented as the mean ± SEM from three independent experiments (*n* = 3). Statistical comparisons were computed by one‐way ANOVA (*P < 0.05, **P < 0.01, ***P < 0.001). (D) Representative western blot images show the changes in the protein expression of AATs, total‐ and phospho‐S6 ribosomal protein in HCT116 and DLD‐1 cells after the shRNA transfection. β‐actin was used as protein loading control. All the experiments were performed three times independently, and the blots are representative of three independent replicates.

As stated above, AATs play important roles in AA‐dependent mTOR signaling and tumor cell growth, since they increase the cellular uptake of AAs across the plasma membrane, for example via members of the SLC1, SLC7, and SLC38 family. In recent years, several reports have shown the tumor growth‐promoting role of SLC1A5/ASCT2 in several cancers. However, the roles of these key AATs, SLC1A5/ASCT2, SLC7A5/LAT1, and SLC38A2/SNAT2 in CRC progression has received little attention. Therefore, to understand the role of these AATs in amino acid uptake, radioisotope‐labeled l‐glutamine and l‐leucine uptake assays were performed in SW480, SW620, HCT116, and DLD‐1 cells following the transfection of shRNAs against AATs. Our data show a significant decrease in the uptake of l‐glutamine in CRC cancer cells following the knockdown of SLC1A5/ASCT2 and SLC38A2/SNAT2, whereas knockdown of SLC7A5/LAT1 did not reveal any significant changes in the l‐glutamine uptake (Fig. 4A). However, l‐leucine uptake was reduced following the knockdown of all three AATs (SLC1A5/ASCT2, SLC7A5/LAT1, and SLC38A2/SNAT2), indicating that these AATs collectively participate in the uptake and maintenance of intracellular l‐leucine levels (Fig. 4B). To further address the impact of the knockdown of these AATs on CRC cell proliferation, XTT cell proliferation assays were performed for up to 5 days. Our results reveal that the knockdown of AATs in SW480, SW620, DLD‐1, and HCT116 cells significantly decreases proliferation (Fig. 4C) as well as colony formation (Fig. S4A,B). Furthermore, to understand whether these three selected AATs‐mediated amino acid uptake is linked to mTOR activation, we analyzed the impact of the knockdowns of these AATs on the mTOR pathway. The western blot results show that knockdown of AATs strongly inhibits activation S6 ribosomal protein (pS6), a downstream target of mTOR pathway (Fig. 4D). Collectively, our data show that the selected AATs (SLC1A5/ASCT2, SLC7A5/LAT1, and SLC38A2/SNAT2) play important roles in the activation of mTOR and CRC cell proliferation, by facilitating uptake of amino acids such as l‐glutamine and l‐leucine.

### 3.6 KRAS mutant CRC cells are highly sensitive to pharmacological inhibitors of AATs

**Fig. 5:**
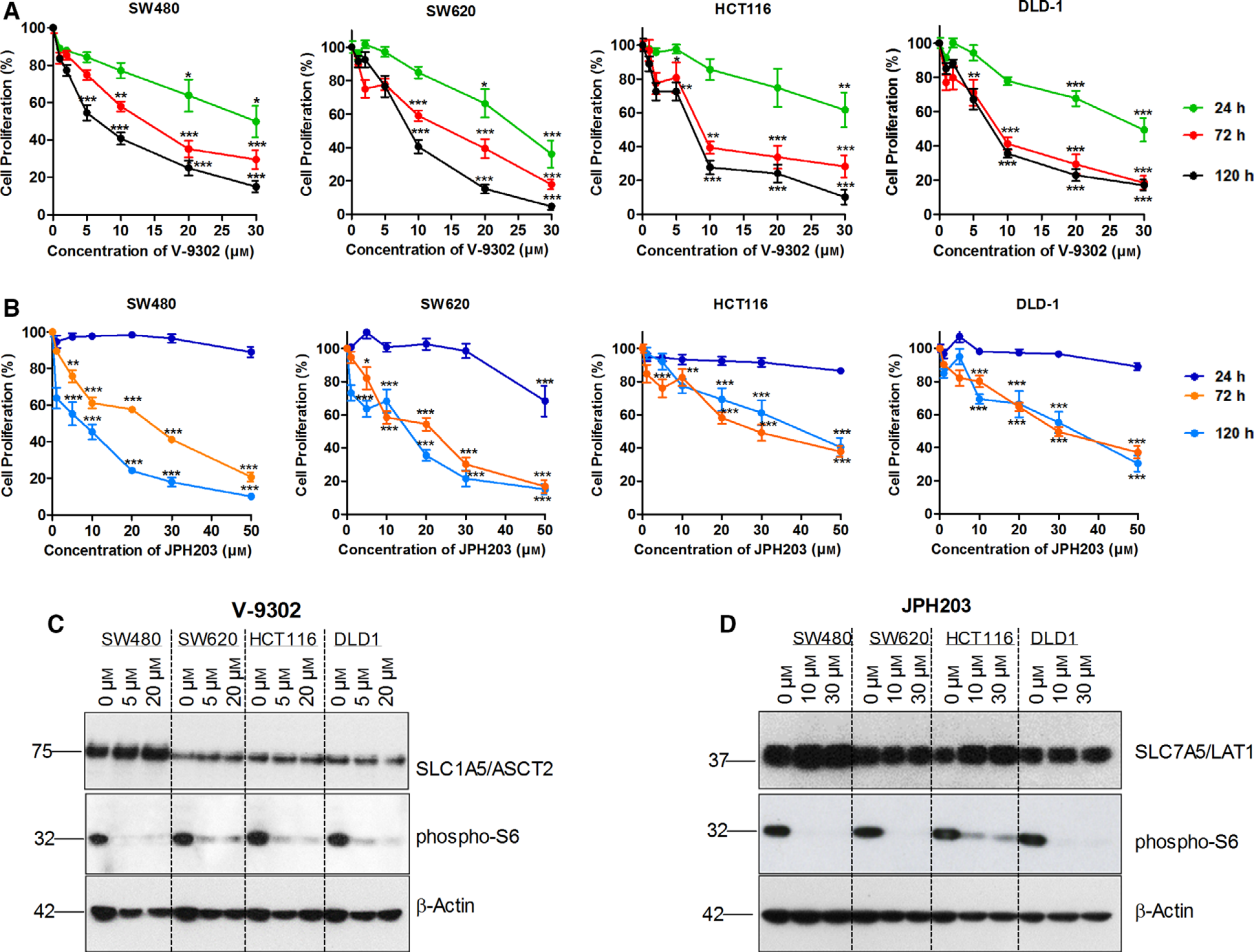
Pharmacological inhibitors of AATs inhibits the CRC cell proliferation and mTOR activation. (A, B) Changes in the proliferation rate of CRC cell lines (SW480, SW620, HCT116, and DLD‐1) following the treatment with different concentration of JPH203 and V‐9302 at 24, 72, and 120 h. Each data point is represented as mean ± SEM. *n* = 3 independent experiments performed in triplicate. *P < 0.05, **P < 0.01, ***P < 0.001 vs vehicle control. (C, D) Representative western blot images show the changes in the activation of ribosomal S6 protein in different CRC cells following the treatment with JPH203 and V‐9302 at 48h. All data are presented as the mean ± SEM from three independent experiments. Western blotting was performed three times independently, and the blots shown are representative of three independent replicates.

Recent reports have shown that the inhibitors of AATs (JPH203 and V‐9302) effectively reduced the proliferation of different cancer cell types by inhibiting mTOR activation. JPH203, a LAT1 specific inhibitor, has been reported to inhibit EAA transport (e.g., leucine) and mTOR activation. On the other hand, V‐9302 has been shown to strongly reduce cancer cell proliferation with combined inhibition of SLC38A2/SNAT2‐ and SLC7A5/LAT1‐mediated amino acid transport [48]. To evaluate the effects of these inhibitors on CRC cell proliferation, we performed XTT cell proliferation assay on KRAS mutant CRC cell lines (SW480, SW620, HCT116, and DLD‐1) and KRAS wt CRC cells (Caco‐2 and HKe3‐KRAS^WT/WT+^) following the treatment with the JPH203 and V‐9302. Our data show a dose‐dependent decrease in the proliferation of CRC cells following the treatment with the pharmacological inhibitors of AATs (Fig. 5A,B and Fig. S5A,B). Interestingly, our results show that KRAS mutant CRC cells are highly sensitive to the pharmacological inhibition of AATs as compared to the KRAS wt CRC cells (Fig. 5A,B). Furthermore, our data reveal strong inhibition of mTOR (phospho‐S6) in KRAS mutant CRC cells (Fig. 5C,D). In contrast, pharmacological inhibition of AATs had less significant effect on the proliferation and mTOR activation of KRAS wt CRC cell lines, Caco‐2, and Hke3‐KRAS^WT/WT+^ (Fig. S5A–D). Collectively, our results indicate that pharmacological inhibitors of AATs have a significant growth inhibitory effect on KRAS mutant CRC cells through inactivation of mTOR.

### 3.7 Role of hippo signaling effector YAP1 in the regulation of AAT expression in CRC cells

To understand the mechanism of regulation of these AATs downstream of oncogenic KRAS, we performed GSEA on publicly available microarray gene expression dataset (GSE119197) of DLD‐1 cells individually expressing isogenic KRAS^G13D^ mutation or KRAS^WT^ oncogene [49]. In addition, we also performed GSEA on an RNA‐sequence dataset of Hke3 cells (GSE110649) individually expressing KRAS^WT/G13D+^ or KRAS^WT/WT+^ oncogenes [23]. Results from GSEA report show that oncogenic signatures such as KRAS, MEK, AKT, and MTOR signaling targets and transcription factors targets (e.g., ATF, E2F) were positively enriched in KRAS mutant CRC cells (Table S3). In addition, GSEA revealed that KRAS mutant CRC cells (DLD‐1‐KRAS^G13D^ and Hke3‐KRAS^WT/13D+^) have significant enrichment of ‘YAP1 driven gene signature’ (Fig. S6A,B; Table S3). Previous reports have shown that oncogenic KRAS promotes cancer cell metabolism and proliferation by altering the AKT, MTOR, E2F, and MYC pathways. However, the involvement of YAP1 in the regulation of AATs, mTOR activation, and cell proliferation in KRAS mutant CRC cells is unknown.

**Fig. 6:**
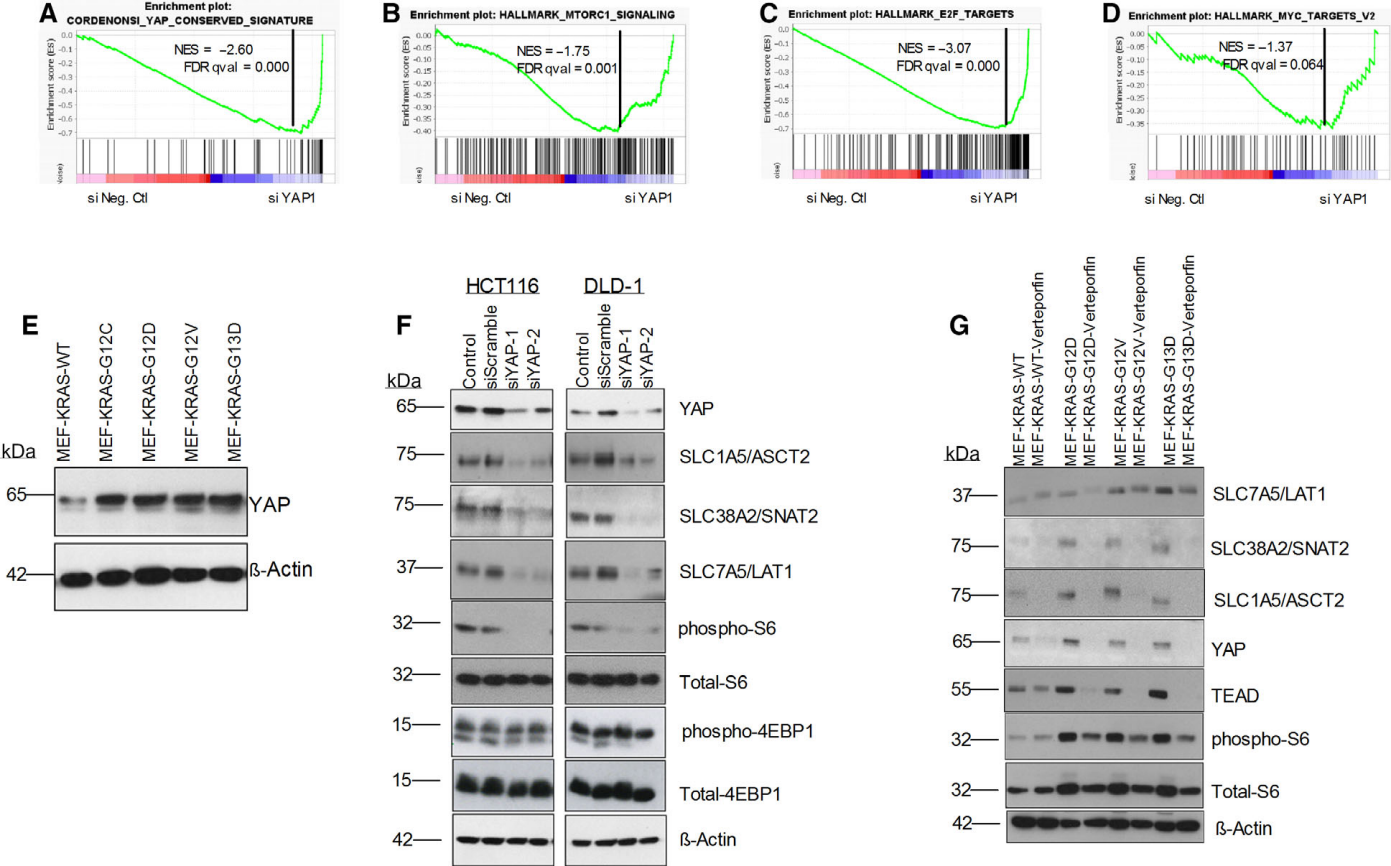
Role of hippo‐pathway effector, YAP1 in the regulation of the expression of AATs in CRC cells: GSEA plot show downregulation of Cordenonsi YAP conserved signature (A), mTORC1 signaling pathway (B), E2F targets (C), and MYC targets (D) in siYAP1 transfected HCT116 cells. (E) Representative western blot images show the protein expression of YAP1 in MEF‐KRAS cells. (F) Changes in the protein expression of AATs, total‐S6, phospho‐S6, total‐4EBP1, phospho‐4EBP1, and YAP1 in HCT116 and DLD‐1 cell lines following the transfection of siRNAs against YAP1. (G) Effect of verteporfin (YAP/TEAD inhibitor) on the expression of AATs, YAP1, TEAD, total‐S6, and phospho‐S6 in MEF‐KRAS cell lines. β‐actin was used as protein loading control. All the experiments were performed three times independently, and the blots are representative of three independent replicates.

To gain further insights into the role of YAP1 in the regulation of AATs and mTOR pathway in KRAS mutant CRC cells, we performed GSEA on publicly available microarray dataset (GSE92335) derived from YAP1 knockdown HCT116 cells [50]. Results from this GSEA show that HCT116 cells upregulated several oncogenic and hallmark pathways, including KRAS, E2F, G2M, mTORC1, and MYC, while YAP1 siRNA‐transfected cells had a significant downregulation of target genes of KRAS, E2F, G2M, MTORC1, and MYC pathways (Fig. 6A–D; Tables S4 and S5). From these results, we assumed that YAP1 may be required, downstream of KRAS, for supporting the MYC/E2F‐driven gene expression of AATs, mTOR activation, and cell proliferation.

To validate these findings, we first tested the abundance of YAP1 protein levels in KRAS mutant MEF cells, by western blotting. Interestingly, protein expression of transcriptional coactivator YAP1 was significantly upregulated in KRAS mutant MEFs compared with wt KRAS‐expressing MEF cells (Fig. 6E). In support of these findings, using TCGA CRC patient datasets from the cBioportal Cancer genome database [51, 52], we found significant positive correlation of YAP1 mRNA levels in KRAS mutant CRC samples (Fig. S6C). To validate further whether YAP1 is critical for regulation of expression of AATs, siRNAs specifically targeting YAP1 were transfected into CRC cells and the changes in expression of AATs were analyzed by western blotting. Our data show that siRNA‐mediated knockdown of YAP1 resulted in a dramatically decreased expression of the AATs and mTOR activation in CRC cell lines HCT116 and DLD‐1 (Fig. 6F). Furthermore, we show that the small molecule inhibitor of YAP1/TEAD interaction, verteporfin treatment downregulates the expression of AATs (SLC7A5/LAT1, SLC38A2/SNAT2, and SLC1A5/ASCT2), and mTOR activation in MEFs expressing mutant KRAS oncogenes (Fig. 6G). Altogether, these data demonstrate that the hippo signaling effector YAP1, upstream of TFs (e.g., MYC and E2F), is critical for regulation of AATs expression in KRAS mutant CRC cells.

### 3.8 Overexpression of AATs in CRC patient tissues and their association with KRAS mutation status and poor patient survival

**Fig. 7:**
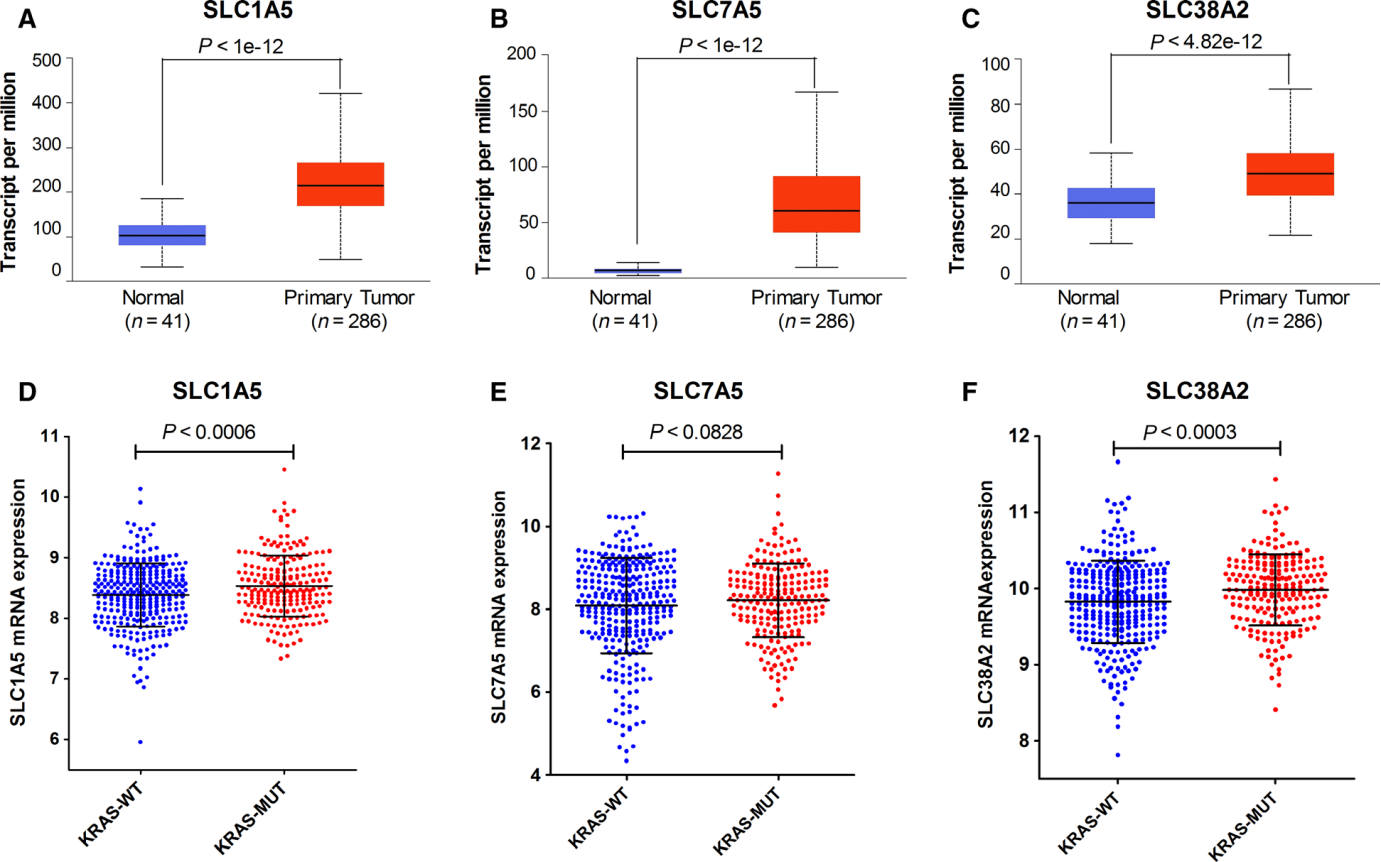
Upregulation of AATs in CRC patient samples and their association with oncogenic KRAS mutations: (A–C) Box‐whisker plots show mRNA expression levels of AATs (SLC1A5, SLC7A5, and SLC38A2) in TCGA colon adenocarcinoma patient samples (*n* = 286) and normal samples (*n* = 41). Box‐whisker plots represent the interquartile range, middle line indicates the median, and the whiskers indicate minimum/maximum values. (D–F) Correlation of mRNA expression levels of AATs (SLC1A5, SLC7A5, and SLC38A2) in CRC patient samples harboring KRAS wt (*n* = 317) and KRAS mutations (*n* = 217), retrieved from gene expression omnibus database (NCBI‐GEO). Error bars represent mean ± SEM; *P*‐values were calculated by unpaired *t*‐test.

In several previous reports, AATs expressions have been shown to be upregulated in multiple cancer types and poor patient survival. However, there has been no detailed report showing that the expression of this specific set of AATs (SLC1A5, SLC7A5, and SLC38A2) is associated with KRAS oncogenic mutations. Therefore, to establish the expression pattern of AATs as biomarkers for CRCs, firstly, we found significant upregulation of these AATs (SLC1A5/ASCT2, SLC7A5/LAT1, and SLC38A2/SNAT2) in CRC patient tissues (*n* = 286) as compared to normal tissue samples (*n* = 41) using TCGA colon adenocarcinoma (COAD) gene expression dataset from the UALCAN database (Fig. 7A–C). Furthermore, to substantiate whether the expression levels of these AATs are linked to the KRAS mutation status in human CRC, we analyzed the gene expression levels of AATs in a large, publicly available CRC cohort (> 580 CRC tumors) at the GEO database. In line with our *in vitro* findings, gene expression analysis showed that CRC tumor specimens from patients harboring KRAS mutations (*n* = 217) display significantly higher mRNA expression levels of AATs compared with KRAS WT patients (*n* = 317) (Fig. 7D–F). Furthermore, to corroborate whether upregulation of these AATs associated with KRAS mutations, we analyzed a RNA‐sequencing dataset of mouse intestinal organoids harboring endogenous KRAS^G12D^ and APC mutations. Interestingly, the result show upregulation of all three AATs (SLC7A5/LAT1, SLC38A2/SNAT2, and SLC1A5/ASCT2) in mouse intestinal organoids expressing KRAS mutation but not in APC mutant intestinal organoids (Fig. S7A–C). Taken together, these gene expression data suggest that the expressions of AATs (SLC7A5/LAT1, SLC38A2, and SLC1A5) are elevated in KRAS mutant CRC patient tissues and mouse intestinal organoids expressing the endogenous KRAS mutation. Furthermore, Kaplan–Meier survival plot reveals that high levels of AATs (SLC7A5/LAT1, SLC38A2/SNAT2, and SLC1A5/ASCT2) expression correlate with poor clinical outcomes of patients with CRC (Fig. S7D). Collectively, these data reveal a linkage between the expression levels of these three selected AATs (SLC7A5/LAT1, SLC38A2/SNAT2, and SLC1A5/ASCT2) and the oncogenic KRAS mutation status in patients with CRC.

## 4 Discussion

Blocking the accelerated uptake of specific nutrients such as glucose and amino acids in cancer cells is considered to be a valuable therapeutic strategy to starve cancer cells and to prevent tumor growth that is safer than classical forms of treatments such as cytotoxic anticancer chemotherapy [6, 14, 19]. Most of such nutrients are delivered at high concentrations across the plasma membrane to the cancer cells via SLC solute carriers (e.g., glucose and amino acid transporters) and, indeed, the expression of several of these transporters is markedly upregulated in several cancer types [53]. Oncogenic mutations are thought to allow cancer cells to acquire high amount of nutrients from the extracellular milieu through global alterations of gene expression patterns, exploiting the surrounding microenvironment in a corruptive way to actively support tumor growth and dissemination [54, 55]. Many of the previous studies on this topic have focused on the metabolism of glucose and glutamine. More recently, the importance of leucine and arginine and other amino acids have become apparent as well. We are just beginning to understand the mechanisms and extent by which these nutrients modify key metabolic pathways responsible for sustaining tumor energy metabolism and biomass production. In general, the specific AATs that are involved in uptake/efflux of the aforementioned AAs are not well described in the different cancer subtypes. Interestingly, a recent study showed that inhibition of SLC1A5/ASCT2 significantly reduced glutamine uptake in a human breast cancer cell line in a subtype‐dependent manner, with suppression of mTOR signaling, cell growth, and cell cycle progression [12]. In the present study, we demonstrate that oncogenic KRAS mutations promote CRC cell proliferation via upregulation of specific AATs, leading to enhanced amino acid transport and mTOR activation. This reveals a promising approach for the treatment of specific CRC subtypes via modulation of mTOR signaling pathway.

Oncogenic KRAS causes transformation only after activation of upstream stimulants that generate strong and prolonged RAS activity [24, 56]. This activity is critical for reprogramming glucose, glutamine, and nucleotide metabolism to sustain tumor growth [55, 57]. However, the identity of prominent transporters that supply specific nutrients in KRAS mutant tumors is still poorly understood. In addition, it was not clear whether increased transport of AAs in cancer cells correlates with the expression of specific AATs. Our study reveals that KRAS mutations in CRC cells correlate with the higher levels of AA uptake compared with KRAS wt CRC cells (Fig. 1A,B). Interestingly, we show that increased AA uptake is linked to the actual upregulation of specific AATs such as SLC7A5/LAT1, SLC1A5/ASCT2, and SLC38A2/SNAT2 in KRAS mutant CRC cells (Fig. 1C,D). Furthermore, we show that increased AA uptake and consumption accelerates proliferation and colony formation in KRAS mutant cells (Fig. S1A,B), highlighting the importance of enhanced AA uptake in these cells. Consistent with this, a previous report showed upregulation of SLC1A5/ASCT2 and glutamine metabolism genes in an isogenic KRAS mutant CRC cells [21], and another study revealed that the expression of the cystine‐glutamate exchanger SLC7A11/xCT is enhanced by KRAS mutations in different human cancers to cope with resulting oxidative stress [58].

Consistent with these observations, knockdowns of oncogenic KRAS mutations significantly downregulated the expression of AATs (SLC7A5/LAT1, SLC1A5/ASCT2, and SLC38A2/SNAT2), mTOR activation and cell proliferation by decreasing the l‐glutamine and l‐leucine uptake levels in CRC cells (Fig. 2A–C and Fig. S2). Notably, in addition to the knockdowns of oncogenic KRAS, overexpression of mutant KRAS oncogenes also strongly induced the upregulation of these AATs, AA uptake, mTOR activation (phospho‐S6), and cell proliferation in KRAS wt CRC cells (Fig. 3A–E). Furthermore, upregulation of AATs, increased AA uptake, and proliferation was evident in MEFs expressing different common KRAS mutations found in patients with CRC (Fig. S3A–F). This points that KRAS‐mediated regulation of AATs is crucial for the uptake of AAs, mTOR activation, and cancer cell proliferation. Taken together, our data indicate that upregulation of these AATs by oncogenic KRAS mutations is evident in multiple *in vitro* cellular models of KRAS transformation.

Glutamine transport via the members of SLC1, SLC7, and SLC38 is important not only for protein synthesis but also to supply EAAs (e.g., leucine) via SLC7‐mediated amino acid exchange, which is essential to sustain mTORC1 activity in cancer cells [1]. Our data show a significant decrease in l‐glutamine and l‐leucine uptake levels following the knockdown of SLC1A5/ASCT2, SLC7A5/LAT1, and SLC38A2/SNAT2, indicating that these transporters fulfills the AA requirements of CRC cells in a coordinated fashion. In addition, pharmacological inhibitors of AATs (JPH203 and V‐9302) as well as specific knockdowns of SLC1A5/ASCT2, SLC7A5/LAT1, and SLC38A2/SNAT2 significantly reduced amino acid transport, which decreased CRC proliferation via inhibition of mTOR activation (Figs. 4A–D and 5A–D). Notably, pharmacological inhibitors of AATs showed a significant anti‐proliferative effects on KRAS mutant CRC cells, compared with KRAS wt cells (Fig. 5A,B and Fig. S5A,B). This demonstrates a genotype specific susceptibility and indicates that inhibition of these selected AATs may offer therapeutic benefit for the CRC patients with KRAS mutations.

As shown previously, mTOR regulates cancer cell metabolism by controlling expression of the TFs c‐Myc and HIF1α [2]. The importance of c‐Myc in cancer metabolism has been reported in many cancer cells [59]. However, the key upstream regulators of TFs (e.g., MYC and E2F) involved in the regulation of AATs in cancer cells have not been reported yet. Herein, we report that hippo signaling pathway effector YAP1 is involved in the regulation of expression of AATs in KRAS mutant CRC cells (Fig. 6A–G and Fig. S6A–C). The hippo signaling pathway, consisting of a highly conserved downstream transcription coactivators (YAP1 and TAZ), plays a key role in tissue homeostasis and organ size control by regulating tissue‐specific stem cells. Moreover, this pathway plays a prominent role in tissue repair and regeneration. Dysregulation of the hippo‐pathway is known to be associated with cancer development as well. Recent evidences showed that YAP1 is necessary downstream of mutant KRAS, to enable the expression of genes regulated by KRAS signaling [60]. Loss of YAP1 in epithelial cells also reduced the number of tumor promoting cancer‐associated fibroblasts and lymphocytes in early stage lesions, suggesting that YAP1 influences KRAS‐mediated tumor progression through multiple mechanisms. In line with these previous reports, our GSEA results show that YAP‐driven gene signature is highly enriched in KRAS mutant CRC cells, HCT116, and DLD‐1 (Fig. S6A,B). Interestingly, knockdown of YAP1 in KRAS mutant CRC cells (HCT116) showed downregulation of MYC and E2F TFs targets as well as mTOR pathway (Fig. 6A–D).

Furthermore, our data indicate that enhanced expression of AATs and amino acid transport in to CRC cells is linked to the upregulation and stabilization of the transcriptional coactivator YAP1 in KRAS mutant CRC cells. Consistent with this, overexpression of oncogenic KRAS mutations in MEFs showed increased expression/abundance of YAP1 protein level (Fig. 6E). In addition, YAP1 abundance was associated with KRAS mutations in patients with CRC (Fig. S6C). Furthermore, specific knockdown of YAP1 and pharmacological inhibitor of YAP1 (verteporfin) inhibited the AATs expression and mTOR activation (phospho‐S6 and 4EBP1) in CRC cells and MEFs (Fig. 6F,G). Collectively, these findings improve our understanding of how exactly KRAS mutant cancer cells upregulate expression of AATs as upstream stimulants, which is clinically relevant, since they are overexpressed in multiple cancers and therefore represent attractive therapeutic targets.

Eventually, our *in vitro* findings are reinforced by the analysis of the TCGA gene expression datasets, showing that selected AATs (SLC1A5/ASCT2, SLC7A5/LAT1, and SLC38A2/SNAT2) are significantly upregulated in primary colorectal tumor tissues, compared with normal tissues (Fig. 7A–C). Interestingly, in line with our *in vitro* findings, gene expression analyses on publicly available CRC patient clinical datasets and mouse intestinal organoids derived from a preclinical CRC mouse model reveal KRAS mutation‐dependent upregulation of the selected AATs (Fig. 7D–F and Fig. S7A–C, respectively). Furthermore, our data highlight overexpression of AATs (SLC1A5/ASCT2, SLC7A5/LAT1, and SLC38A2/SNAT2) as prognostic marker of poor survival of patients with CRC (Fig. S7D). Thus, our data unveil the upregulation of the selected AATs (SLC1A5/ASCT2, SLC7A5/LAT1, and SLC38A2/SNAT2) as a prognostic marker of poor survival of patients with CRC, useful for cancer patient stratification, in particular with regard to the KRAS mutant subtype of patients.

## 5 Conclusions

Overall, our data demonstrate that oncogenic KRAS trigger upregulation of specific AATs (SLC7A5/LAT1, SLC1A5/ASCT2, and SLC38A2/SNAT2) via hippo signaling effector YAP1, leading to mTOR activation and CRC cell proliferation. The hippo‐pathway effector YAP1 is likely involved in the regulation of these AATs downstream of mutant KRAS in CRC cells. These selected AATs thus represent attractive candidate therapeutic targets for cancer treatment. Future studies with all known SLC‐nutrient transporters using patient‐derived cancer tissue specimens will be important to further expand our knowledge in this field.

## Conflict of interest

The authors declare no conflict of interest.

## Author contributions

PK, IZ and MAH originally conceptualized, designed the study, and prepared the manuscript. PK performed the experiments, analyzed the data, and generated final figures presented in this manuscript. DTN, JPG, and RB provided administrative, technical, and materials support. MAH supervised the study. All authors have read and approved the final manuscript.

### Peer Review

The peer review history for this article is available at https://publons.com/publon/10.1002/1878‐0261.12999.

## Supporting information

**Fig. S1.** KRAS mutant CRC cells dependence on glutamine for their proliferation.

**Fig. S2.** Knockdown of oncogenic KRAS inhibits CRC cell proliferation and colony formation.

**Fig. S3.** Overexpression of KRAS mutations upregulates AATs and increases glutamine dependency in MEFs.

**Fig. S4.** Knockdown of AATs (SLC1A5, SLC7A5, and SLC38A2) inhibits the CRC cell colony‐forming efficiencies.

**Fig. S5.** Effect of pharmacological inhibitors of AATs on HKe3‐KRAS^WT/WT+^ and Caco‐2 cells.

**Fig. S6.** GSEA between KRAS mutant vs KRAS wt CRC cells and association of YAP1 expression with KRAS mutation in patients with CRC.

**Fig. S7.** Upregulation of mRNA expression levels of selected AATs in KRAS mutant mouse intestinal organoids and their association with CRC patient survival probability.

**Table S1.** shRNA primers used in this study.
**Table S2.** Real‐time qPCR primers.
**Table S3.** GSEA result show differentially expressed pathways in Hke3‐KRAS mutant cells.
**Table S4.** GSEA result show downregulation of major hallmark pathways in siYAP‐transfected HCT116 cells.
**Table S5.** GSEA result show downregulation of major oncogenic signaling pathways in siYAP‐transfected HCT116 cells.

**Appendix S1.** Densidometry data for Western blots.

## Data Availability

All raw data are available from the corresponding author upon reasonable request.
