# Supplementary figures and images for: Oncogenic KRAS mutations enhance amino acid uptake by colorectal cancer cells via the hippo signaling effector YAP1
Source: Mol Oncol. 2021 Jun 18;15(10):2782–800. doi: 10.1002/1878-0261.12999 (PMC8486573; doi:10.1002/1878-0261.12999)

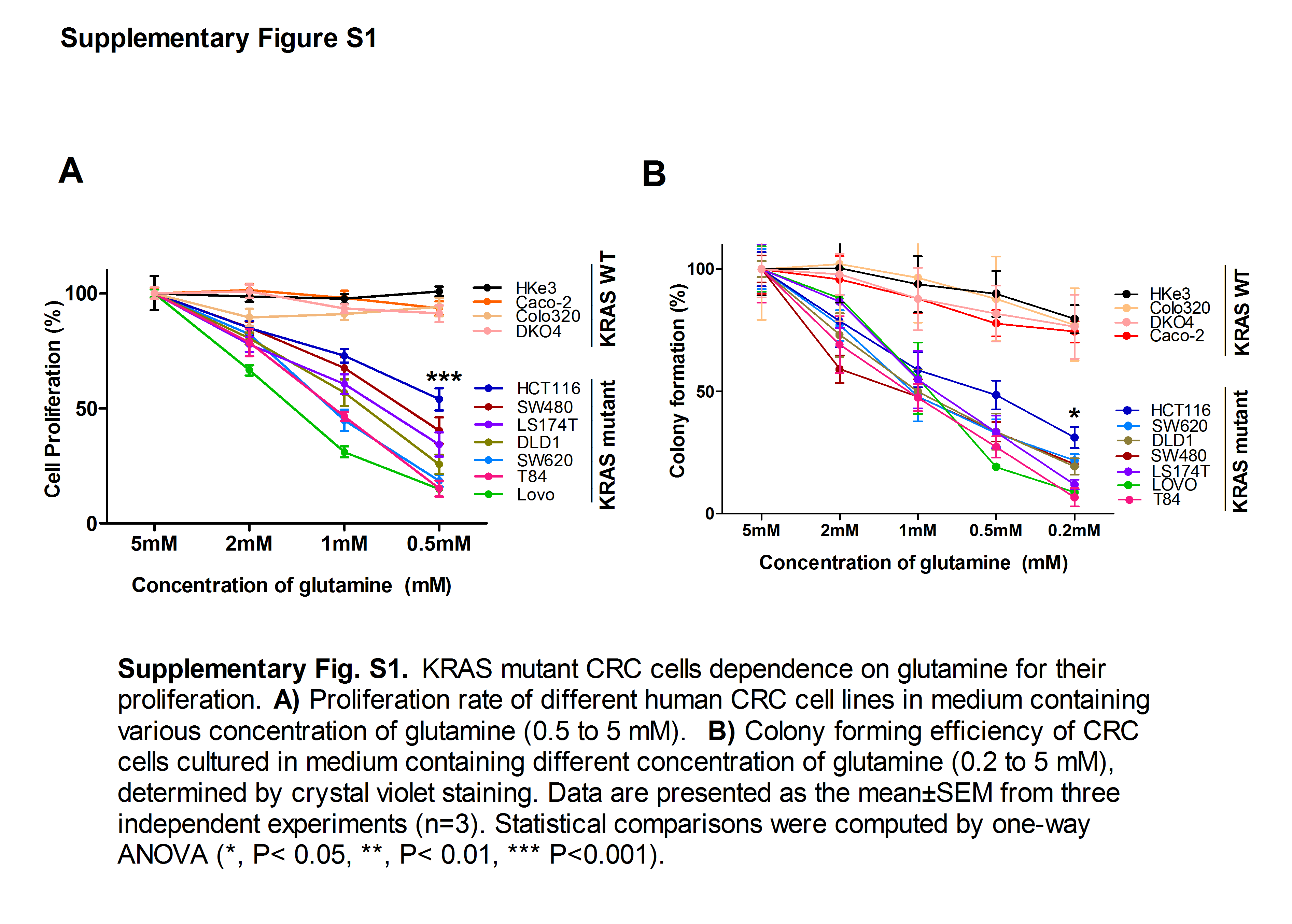

Supplement: Supplementary file 1 — Fig. S1. KRAS mutant CRC cells dependence on glutamine for their proliferation. [file MOL2-15-2782-s004.tif]

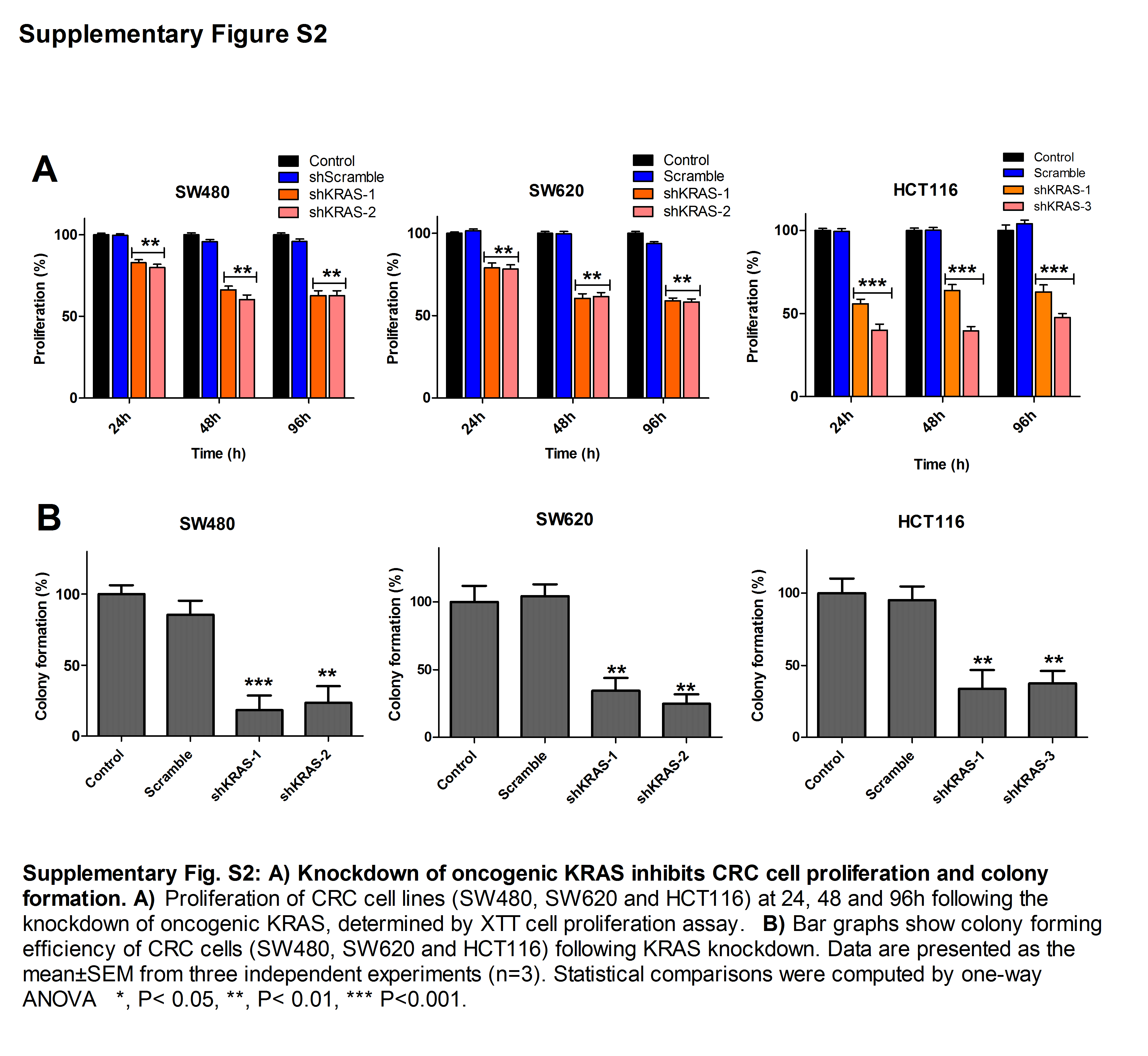

Supplement: Supplementary file 2 — Fig. S2. Knockdown of oncogenic KRAS inhibits CRC cell proliferation and colony formation. [file MOL2-15-2782-s001.tif]

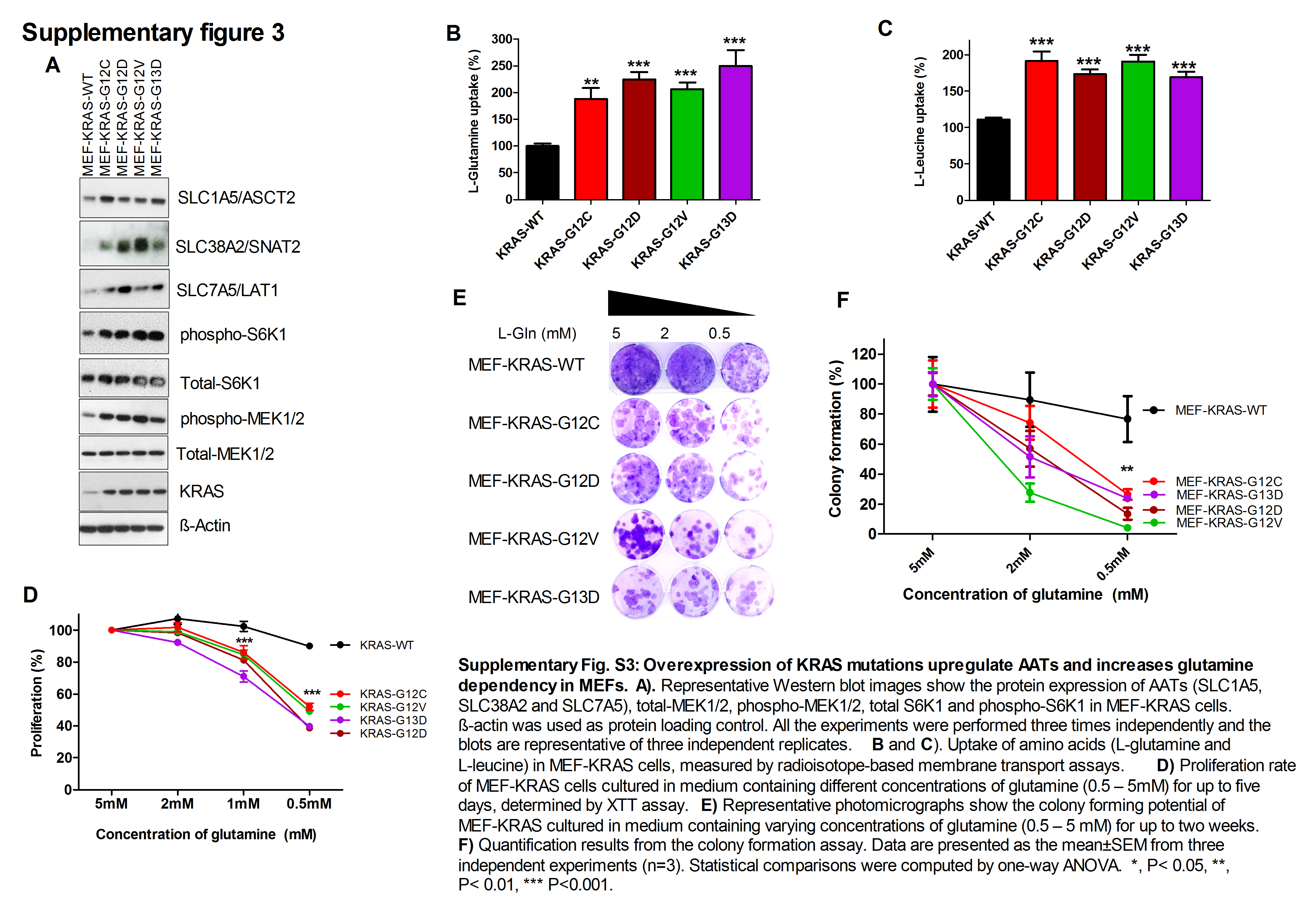

Supplement: Supplementary file 3 — Fig. S3. Overexpression of KRAS mutations upregulates AATs and increases glutamine dependency in MEFs. [file MOL2-15-2782-s003.tif]

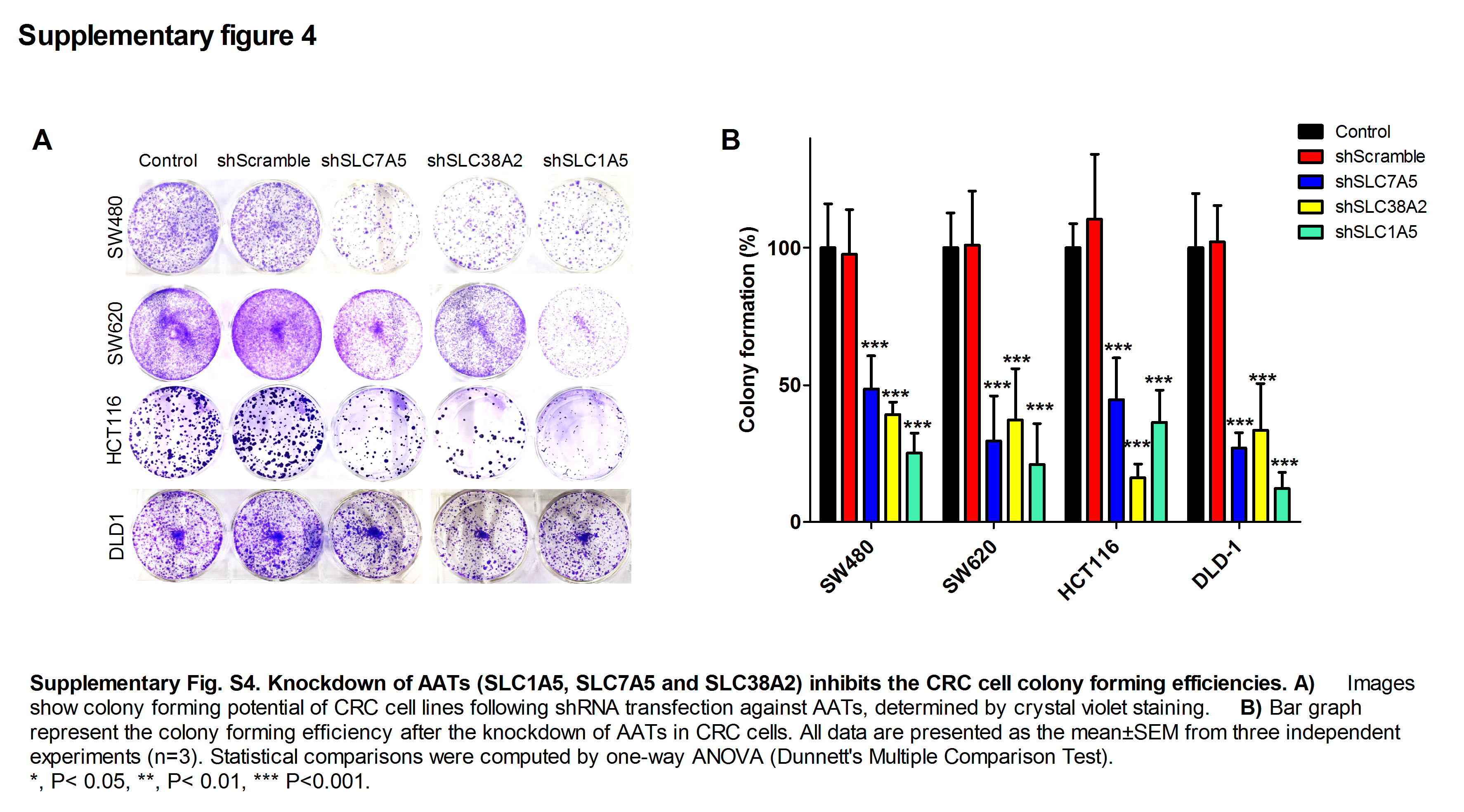

Supplement: Supplementary file 4 — Fig. S4. Knockdown of AATs (SLC1A5, SLC7A5, and SLC38A2) inhibits the CRC cell colony‐forming efficiencies. [file MOL2-15-2782-s007.tif]

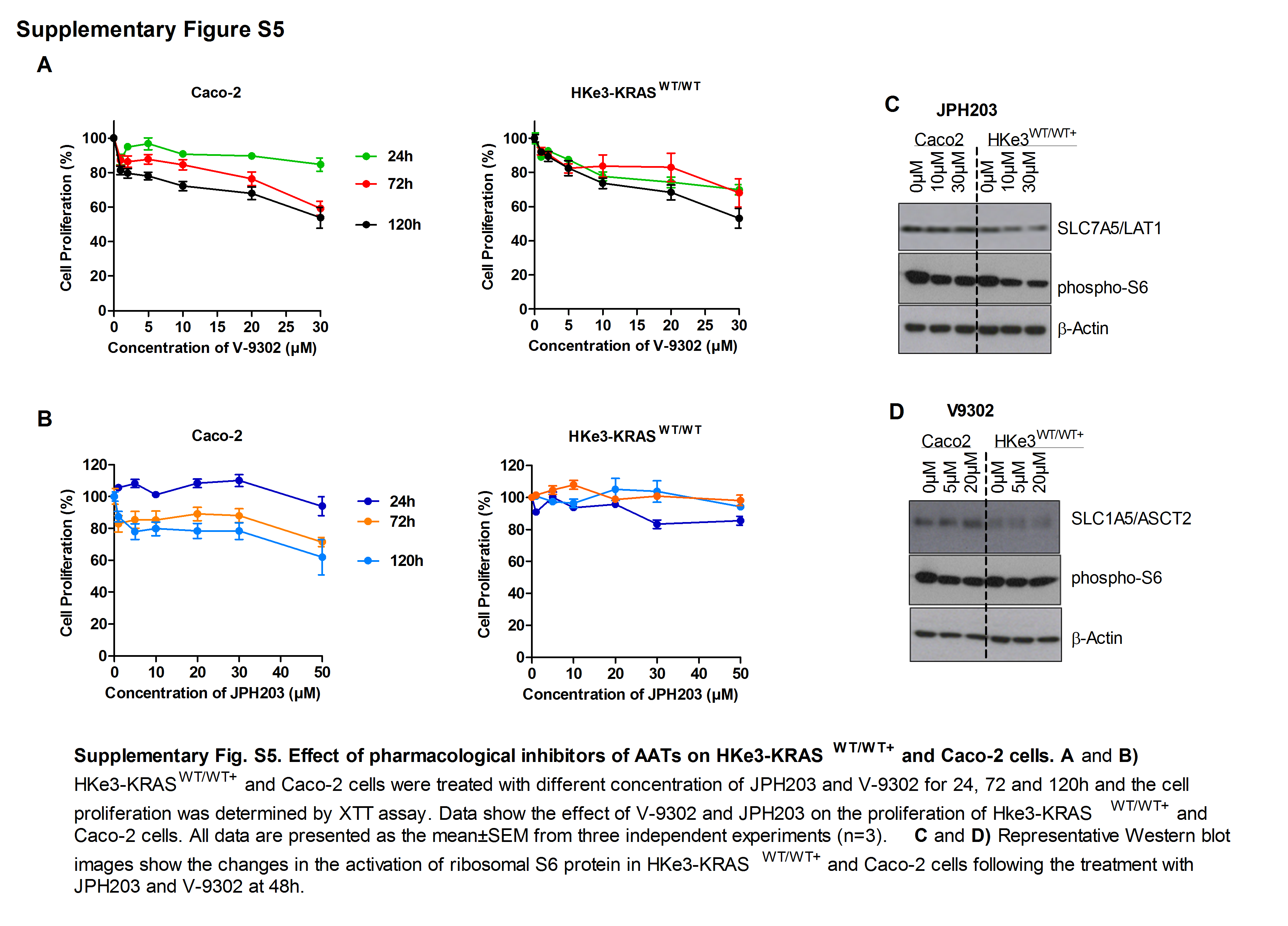

Supplement: Supplementary file 5 — Fig. S5. Effect of pharmacological inhibitors of AATs on HKe3‐KRASWT/WT+ and Caco‐2 cells. [file MOL2-15-2782-s002.tif]

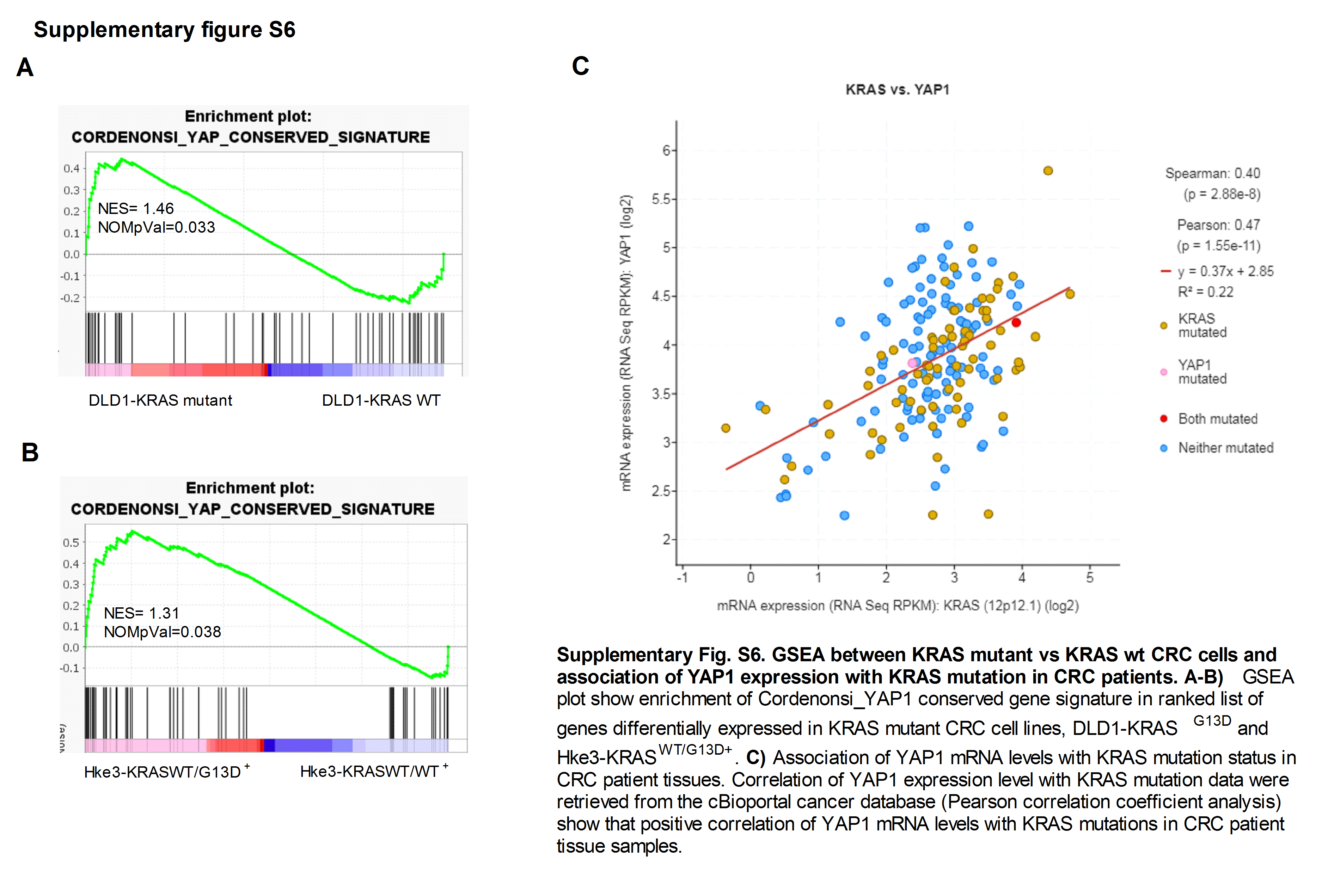

Supplement: Supplementary file 6 — Fig. S6. GSEA between KRAS mutant vs KRAS wt CRC cells and association of YAP1 expression with KRAS mutation in patients with CRC. [file MOL2-15-2782-s009.tif]

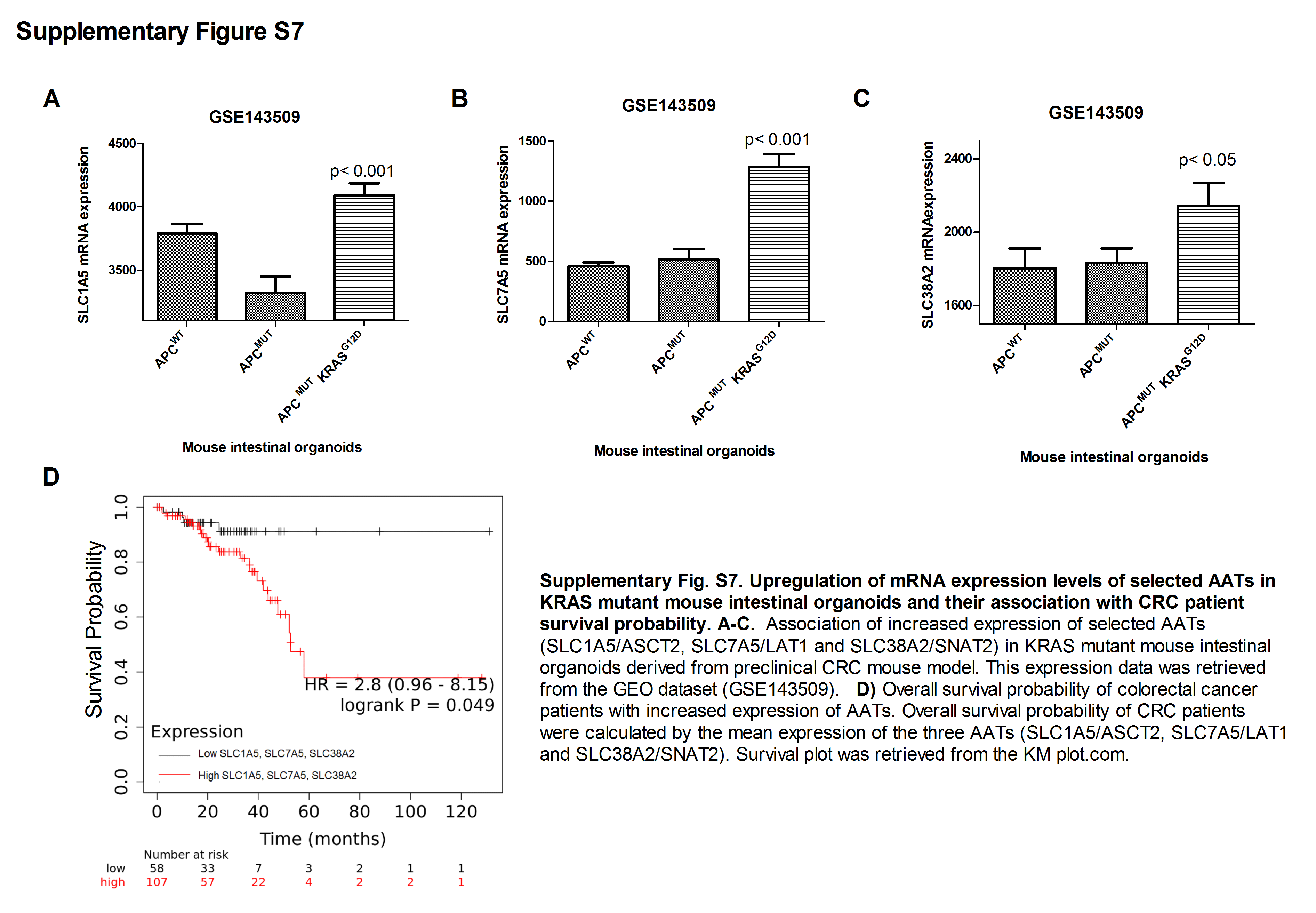

Supplement: Supplementary file 7 — Fig. S7. Upregulation of mRNA expression levels of selected AATs in KRAS mutant mouse intestinal organoids and their association with CRC patient survival probability. [file MOL2-15-2782-s005.tif]
