# Supplementary material for: Oncogenic KRAS mutations enhance amino acid uptake by colorectal cancer cells via the hippo signaling effector YAP1
Source: Mol Oncol. 2021 Jun 18;15(10):2782–800. doi: 10.1002/1878-0261.12999 (PMC8486573; doi:10.1002/1878-0261.12999)
Supplement: Supplementary file 8 — Table S1. shRNA primers used in this study. Table S2. Real‐time qPCR primers. Table S3. GSEA result show differentially expressed pathways in Hke3‐KRAS mutant cells. Table S4. GSEA result show downregulation of major hallmark pathways in siYAP‐transfected HCT116 cells. Table S5. GSEA result show downregulation of major oncogenic signaling pathways in siYAP‐transfected HCT116 cells. [file MOL2-15-2782-s008.pdf]

Supplementary Table 1. shRNA primers used in this study

shKRAS-1	FWD	GATCCCCGTTGGAGCTGATGGCGTAGTTCAAGAGACTACGCCATCAGCTCCAACTTTTTGGAAA
	REV	AGCTTTTCCAAAAAGTTGGAGCTGATGGCGTAGTCTCTTGAACTACGCCATCAGCTCCAACGGG
shKRAS-2	FWD	GATCCCCGTTGGAGCTGTTGGCGTAGTTCAAGAGA CTACGCCAACAGCTCCAACTTTTTGGAAA
	REV	AGCTTTTCCAAAAAGTTGGAGCTGTTGGCGTAGTCTCTTGAACTACGCCAACAGCTCCAACGGG
shKRAS-3	FWD	GATCCCCGTTGGAGCTGGTGACGTAGTTCAAGAGACTACGTCACCAGCTCCAACTTTTTGGAAA
	REV	AGCTTTTCCAAAAAGTTGGAGCTGGTGACGTAGTCTCTTGAACTACGTCACCAGCTCCAACGGG
shLAT1 #1	FWD	GATCCCCGCGGCCGAGGAGAAGGAAGTTCAAGAGACTTCCTTCTCCTCGGCCGCTTTTTGGAAA
	REV	AGCTTTTCCAAAAAGCGGCCGAGGAGAAGGAAGTCTCTTGAACTTCCTTCTCCTCGGCCGCGGG
shSLC38A2 #1	FWD	GATCCCCGGACGATTCAGTATTTCCCTTCAAGAGAGGGAAATACTGAATCGTCCTTTTTGGAAA
	REV	AGCTTTTCCAAAAAGGACGATTCAGTATTTCCCTCTCTTGAAGGGAAATACTGAATCGTCCGGG
shASCT2 #1	FWD	GATCCCCCTGGATTATGAGGAATGGATTCAAGAGATCCATTCCTCATAATCCAGTTTTTGGAAA
	REV	AGCTTTTCCAAAAACTGGATTATGAGGAATGGATCTCTTGAATCCATTCCTCATAATCCAGGGG
Scramble shRNA	FWD	GATCCCCGCGAAAGATGATAAGCTAATTCAAGAGATTAGCTTATCATCTTTCGCTTTTTGGAAA
	REV	AGCTTTTCCAAAAAGCGAAAGATGATAAGCTAATCTCTTGAATTAGCTTATCATCTTTCGCGGG

Supplementary Table 2. Real time qPCR primers

SLC1A2 –FWD - GGTGATGTCAGCTCTCGACG
SLC1A2 – REV – CAGGGAGGGATTGCAAGGTT

SLC1A3 –FWD - TCTTGTTTCTCTGGGGCATCT
SLC1A3 – REV – TTGCTCCAAACAAGCCAAAAC

SLC1A5 – FW - ACCAGGGTGAAGGTGCCCGT
SLC1A5 – REV-CTTCAGGCCCCAGCTTCCGC

SLC1A6 – FWD - GAGGTCGGGAGAACCAAGTG
SLC1A6 –REV – TCCTGCTCAGAAAATGTCCCT

SLC1A7 – FWD - TTGTGGCTTCCCTCTAAGGC
SLC1A7 – REV – CAGGTGGTCGGAGTTGCTAA

SLC3A2 – FWD –ATGCTTGCTGGTGCCGTGGT
SLC3A2 –REV - TTCAGACCCGCCAGGTTGCC

SLC7A1– FW –CTGGCAGCTCACGGAGGAGGA
SLC7A1– REV -GCCCCCGACAGGACACCAGA

SLC7A2 – FW - CTCCTCTTCGCGGGACCA
SLC7A2 – REV- AGACATCGGGCAAAGGTCAG

SLC7A5 – FW –GAGAAGGAAGAGGCGCGG
SLC7A5 – REV -CCGAGCCGATAATGGTCCC

SLC7A6– FW –TGGCTGCTGCTTGCATATGTCTGC
SLC7A6– REV –ATGAGCGCTACGACCTTGGCG

SLC7A7– FW –AGGCATTTGTCTGGCCTTCCCTTT
SLC7A7– REV –TGGCATTGCCTCCTTGGTCCTG

SLC7A8– FW –TCCCCCGCCCCATCAAGATCA
SLC7A8– REV –GTGCAGCAGGAGTGGCTGGG

SLC7A9 – FW - GAGGAAGCCAGCTCCCCTA
SLC7A9 – REV – CCATGTTTCCTCCTGCTGGT

SLC7A11 – FW - ATGGGACAAGAAACCCAGGTG
SLC7A11 – REV – TCCCTATTTTGTGTCTCCCCTTG

SLC38A1 – FW -AGCGGCGGTGCATGTGGA
SLC38A1 – REV –GGAAGCTTGACACCCCTGTTAGC

SLC38A2 – FW - AGCATGAAGAAGGCCGAAAT
SLC38A2 – REV - TTCAGAGCAGCTTGCTTGG

SLC38A3 – FW – GCTGTTGCAGCCGAGTTC

SLC38A3 – REV – CACTGGTCTCACACCAACAGT

SLC38A4 – FW - CCCCACTCACACAGAACAGAG
SLC38A4 – REV – CAGCGCTTTCTTGTCCACAC

SLC38A6 – FW – CAAGTCGACTCGCTCCTCTC
SLC38A6 – REV - TCTACGAGCCTCTACCCCTG

SLC38A7 – FW- TAGATCCCCGAGGTCTGTGG
SLC38A7 – REV – CTGCAGGGAAAGGGTCTGAG

SLC38A8 – FW - CTGACGGGGGTTTATGGCTT
SLC38A8 – REV – GAGCACGATGGGGTAGACAG

SLC38A9 – FW - CCTGTTTGGACTAGGAGCCC
SLC38A9 – REV – CTCGGTGGACCTCAGTTTCC

SLC38A10 –FW- CTGTGAGAGCCAGCATCCAA
SLC38A10 –REV- TGGACACCAAACAGCATCCA

SLC6A14 –FW-ATCAGGACCGTGGTAACTGGTCCAA
SLC6A14 –REV-TCAAGAAGGCGCCTCCACCATTG

SLC6A15 –FW - AAGACACACTGACGGTGCAT
SLC6A15 –REV – TCGCCGAATTCTTTGACCCA

SLC6A18 – FW - GTCACGCTGTCCTTCCTGAT
SLC6A18 – REV – TGTTCTGTTGAGGTCCGGTG

SLC6A19 – FW - AACCTGGTCACAATCGTCCC
SLC6A19 – REV - TCTGCTGCTGTGGAGTCTTG

GAPDH- FW - GGATTTGGTGGTATTGGG
GAPDH- REV - GGAAGATGGTGATGGGATT

Supplementary Table 3: GSEA result show differentially expressed pathways in Hke3-KRAS mutant cells. GSEA summary table show Hke3-KRAS mutant cells upregulated KRAS signaling and several oncogenic pathways, including KRAS, AKT, MEK, MTOR and YAP pathways.

**NAME**	**SIZE**	**ES**	**NES**	**NOM p-val**	**FDR q-val**
KRAS.50_UP.V1_DN	47	0.727041	1.672825	0	0
KRAS.LUNG.BREAST_UP.V1_DN	141	0.590906	1.479416	0	0.029072
LTE2_UP.V1_DN	195	0.577013	1.461095	0	0.027539
KRAS.300_UP.V1_DN	140	0.56784	1.414121	0	0.054492
RPS14_DN.V1_UP	190	0.546408	1.385268	0	0.069131
KRAS.LUNG_UP.V1_DN	142	0.557824	1.379666	0.002	0.061997
CSR_EARLY_UP.V1_DN	126	0.554534	1.376344	0	0.055551
LEF1_UP.V1_DN	187	0.540231	1.360715	0	0.06644
BCAT_GDS748_DN	45	0.591219	1.360629	0.029106	0.059057
PTEN_DN.V2_UP	139	0.555514	1.360524	0.002008	0.053526
KRAS.600.LUNG.BREAST_UP.V1_DN	284	0.531766	1.35987	0	0.049514
MEK_UP.V1_DN	193	0.537247	1.356174	0	0.049454
KRAS.600_UP.V1_DN	279	0.528362	1.35056	0	0.050982
**YAP1_UP**	47	0.576126	1.323469	0.03617	0.083288
AKT_UP.V1_UP	169	0.51491	1.312728	0.002	0.094015
BCAT_BILD_ET_AL_UP	45	0.561913	1.309766	0.048117	0.093536
KRAS.PROSTATE_UP.V1_DN	141	0.521345	1.308069	0.002012	0.090239
DCA_UP.V1_DN	184	0.520673	1.307438	0.002	0.08627
**CORDENONSI_YAP_CONSERVED_SIGNATURE**	57	0.553713	1.306511	0.0375	0.084002
RB_DN.V1_UP	117	0.515256	1.292628	0.022222	0.106851
ESC_V6.5_UP_EARLY.V1_DN	161	0.510946	1.284666	0.006036	0.117957
PKCA_DN.V1_DN	154	0.505047	1.274993	0.016064	0.134621
KRAS.BREAST_UP.V1_DN	143	0.51481	1.26997	0.008048	0.142399
RAPA_EARLY_UP.V1_UP	167	0.502631	1.264955	0.016	0.147812
PRC2_SUZ12_UP.V1_UP	184	0.499434	1.255737	0.004008	0.169384
NOTCH_DN.V1_UP	185	0.490813	1.24712	0.018	0.189612
PDGF_UP.V1_DN	132	0.497363	1.242207	0.030181	0.199217
SNF5_DN.V1_UP	173	0.494283	1.241386	0.018	0.195321
EIF4E_DN	100	0.495009	1.229014	0.085366	0.234694
AKT_UP_MTOR_DN.V1_UP	178	0.483054	1.218779	0.024	0.267937
ATM_DN.V1_UP	146	0.484149	1.215959	0.04	0.271283
CAHOY_ASTROGLIAL	100	0.492037	1.20911	0.092929	0.2952
IL2_UP.V1_DN	192	0.47425	1.201629	0.044177	0.324817
CYCLIN_D1_UP.V1_DN	190	0.474798	1.195467	0.042	0.347847
CAHOY_ASTROCYTIC	100	0.487623	1.194946	0.120724	0.340649
IL15_UP.V1_DN	185	0.470222	1.187365	0.054	0.370684
TBK1.DN.48HRS_UP	50	0.512096	1.186988	0.174004	0.363303
JAK2_DN.V1_DN	144	0.475617	1.184571	0.068273	0.366844
NRL_DN.V1_UP	136	0.473472	1.18423	0.088	0.359507
ALK_DN.V1_DN	139	0.473899	1.183517	0.100402	0.354321
IL21_UP.V1_DN	186	0.467553	1.18291	0.066	0.348241
CSR_LATE_UP.V1_DN	147	0.470532	1.179778	0.090361	0.356131
RAPA_EARLY_UP.V1_DN	184	0.464849	1.174293	0.086	0.377339
CRX_NRL_DN.V1_UP	140	0.467575	1.173311	0.118952	0.373372
PTEN_DN.V1_UP	185	0.468173	1.171977	0.076	0.372629
KRAS.DF.V1_DN	192	0.458927	1.168303	0.07014	0.384446
PRC1_BMI_UP.V1_DN	187	0.462202	1.168163	0.068	0.376907
MTOR_UP.N4.V1_DN	183	0.464254	1.168026	0.078	0.369604
VEGF_A_UP.V1_UP	195	0.462448	1.166695	0.082164	0.368077

Supplementary Table 4: GSEA result show downregulation of major Hallmark pathways in siYAP transfected HCT116 cells. GSEA summary table show HCT116 cells upregulated mTORC1 signaling and several oncogenic pathways, including E2F, G2M, and MYC, while YAP siRNA transfected cells had a significant downregulation of genes in the E2F, G2M, MTORC1 and MYC pathways.

NAME	SIZE	ES	NES	NOM p-val	FDR q-val
HALLMARK_E2F_TARGETS	199	-0.69647	-3.06596	0	0
HALLMARK_G2M_CHECKPOINT	199	-0.6802	-3.0027	0	0
HALLMARK_MITOTIC_SPINDLE	198	-0.54339	-2.34664	0	0
HALLMARK_INTERFERON_ALPHA_RESPONSE	96	-0.55896	-2.23639	0	0
HALLMARK_DNA_REPAIR	150	-0.47329	-2.00096	0	0
HALLMARK_INTERFERON_GAMMA_RESPONSE	200	-0.44235	-1.92045	0	0
HALLMARK_ESTROGEN_RESPONSE_LATE	199	-0.43515	-1.91705	0	0
HALLMARK_UV_RESPONSE_UP	158	-0.44206	-1.88693	0	1.45E-04
HALLMARK_P53_PATHWAY	199	-0.41436	-1.81642	0	5.15E-04
HALLMARK_UNFOLDED_PROTEIN_RESPONSE	112	-0.43743	-1.7759	0.001368	8.48E-04
HALLMARK_UV_RESPONSE_DN	144	-0.42068	-1.77492	0	7.71E-04
HALLMARK_MTORC1_SIGNALING	199	-0.40217	-1.75352	0	8.86E-04
HALLMARK_ESTROGEN_RESPONSE_EARLY	198	-0.38413	-1.68139	0	0.003781
HALLMARK_CHOLESTEROL_HOMEOSTASIS	73	-0.43555	-1.65951	0	0.004252
HALLMARK_APOPTOSIS	161	-0.38847	-1.65605	0	0.003969
HALLMARK_GLYCOLYSIS	200	-0.37827	-1.65468	0	0.003721
HALLMARK_EPITHELIAL_MESENCHYMAL_TRANSITION	200	-0.35431	-1.54489	0	0.013719
HALLMARK_ANDROGEN_RESPONSE	100	-0.3693	-1.46835	0.015537	0.028393
HALLMARK_TNFA_SIGNALING_VIA_NFKB	198	-0.32999	-1.45419	0.007692	0.03105
HALLMARK_HYPOXIA	200	-0.32806	-1.4411	0.002497	0.034345
HALLMARK_MYC_TARGETS_V1	200	-0.3252	-1.42694	0.006361	0.037397
HALLMARK_SPERMATOGENESIS	135	-0.34267	-1.42005	0.01626	0.038783
HALLMARK_MYC_TARGETS_V2	58	-0.36894	-1.36655	0.071207	0.063522
HALLMARK_HEME_METABOLISM	200	-0.30494	-1.33308	0.034527	0.084506
HALLMARK_KRAS_SIGNALING_UP	199	-0.30061	-1.31887	0.02799	0.091213
HALLMARK_IL2_STAT5_SIGNALING	197	-0.29384	-1.29437	0.046572	0.108145

Supplementary Table 5. GSEA result show downregulation of major oncogenic signalling pathways in siYAP transfected HCT116 cells. GSEA summary table show HCT116 cells upregulated Cordenonsi_YAP_conserved_signature, E2F, EGFR, mTORC1 signaling and several oncogenic pathways, including MEK and MYC, while YAP siRNA transfected cells had a significant downregulation of genes in the Cordenonsi YAP conserved signature, MEK, E2F, MTOR and MYC oncogenic pathways.

NAME	SIZE	ES	NES	NOM p-val	FDR q-val	FWER p-val
CORDENONSI_YAP_CONSERVED_SIGNATURE	57	-0.70353	-2.59526	0	0	0
RPS14_DN.V1_DN	186	-0.55987	-2.43561	0	0	0
CSR_LATE_UP.V1_UP	163	-0.56055	-2.41421	0	0	0
RB_P107_DN.V1_UP	129	-0.57326	-2.36787	0	0	0
E2F1_UP.V1_UP	188	-0.52359	-2.28718	0	0	0
E2F3_UP.V1_UP	185	-0.50543	-2.20597	0	0	0
VEGF_A_UP.V1_DN	193	-0.49335	-2.17613	0	0	0
TBK1.DF_UP	286	-0.47382	-2.17589	0	0	0
PRC2_EZH2_UP.V1_DN	191	-0.49764	-2.17092	0	0	0
GCNP_SHH_UP_LATE.V1_UP	178	-0.46995	-2.02557	0	9.72E-05	0.001
PRC2_EED_UP.V1_DN	193	-0.45341	-1.96481	0	8.83E-05	0.001
LTE2_UP.V1_DN	195	-0.44528	-1.95101	0	8.10E-05	0.001
SIRNA_EIF4GI_DN	97	-0.47526	-1.89752	0	3.80E-04	0.005
RAF_UP.V1_UP	193	-0.43187	-1.88164	0	4.17E-04	0.006
TBK1.DN.48HRS_DN	49	-0.52467	-1.85378	0	5.14E-04	0.008
EGFR_UP.V1_DN	196	-0.42149	-1.83506	0	7.79E-04	0.013
TBK1.DF_DN	284	-0.40633	-1.8347	0	7.33E-04	0.013
HOXA9_DN.V1_DN	189	-0.42084	-1.82853	0	8.50E-04	0.016
GCNP_SHH_UP_EARLY.V1_UP	168	-0.42573	-1.82342	0	8.05E-04	0.016
ERBB2_UP.V1_DN	197	-0.39968	-1.76638	0	0.001574	0.033
P53_DN.V1_UP	192	-0.40365	-1.74736	0	0.001869	0.041
CAMP_UP.V1_UP	195	-0.40009	-1.74202	0	0.001913	0.044
EGFR_UP.V1_UP	189	-0.40217	-1.73773	0	0.00196	0.047
MEK_UP.V1_DN	192	-0.39162	-1.71497	0	0.002561	0.064
LTE2_UP.V1_UP	187	-0.3932	-1.70345	0	0.00308	0.08
PTEN_DN.V2_UP	137	-0.40842	-1.70028	0	0.003032	0.08
RB_DN.V1_UP	117	-0.40978	-1.68945	0.001383	0.003358	0.091
KRAS.DF.V1_UP	190	-0.39158	-1.68098	0	0.003544	0.099
AKT_UP.V1_UP	167	-0.3894	-1.66847	0	0.00399	0.114
SNF5_DN.V1_UP	170	-0.38692	-1.66487	0	0.003955	0.117
STK33_DN	276	-0.36071	-1.65047	0	0.004616	0.139
RAF_UP.V1_DN	192	-0.3743	-1.64854	0	0.004502	0.139
RB_P130_DN.V1_UP	127	-0.39573	-1.64136	0	0.004689	0.15
BMI1_DN.V1_UP	145	-0.39015	-1.64036	0	0.004612	0.152
MEK_UP.V1_UP	195	-0.36772	-1.61756	0	0.005899	0.195
EIF4E_DN	100	-0.39046	-1.56786	0.001399	0.010991	0.335
MTOR_UP.N4.V1_UP	194	-0.3589	-1.5646	0	0.011169	0.35
LEF1_UP.V1_UP	191	-0.3582	-1.55002	0.001321	0.012911	0.407
STK33_NOMO_DN	276	-0.34082	-1.54662	0	0.013058	0.416
MTOR_UP.V1_UP	164	-0.36071	-1.53829	0.002663	0.014223	0.45
CAHOY_OLIGODENDROCUTIC	96	-0.38781	-1.5323	0.01273	0.014851	0.472
BCAT_GDS748_UP	48	-0.41912	-1.48121	0.041411	0.026466	0.681
