# Supplementary material for: Oncogenic KRAS mutations enhance amino acid uptake by colorectal cancer cells via the hippo signaling effector YAP1
Source: Mol Oncol. 2021 Jun 18;15(10):2782–800. doi: 10.1002/1878-0261.12999 (PMC8486573; doi:10.1002/1878-0261.12999)
Supplement: Supplementary file 9 — Appendix S1. Densidometry data for Western blots. [file MOL2-15-2782-s006.pdf]

Figure 1D. Western blot quantification

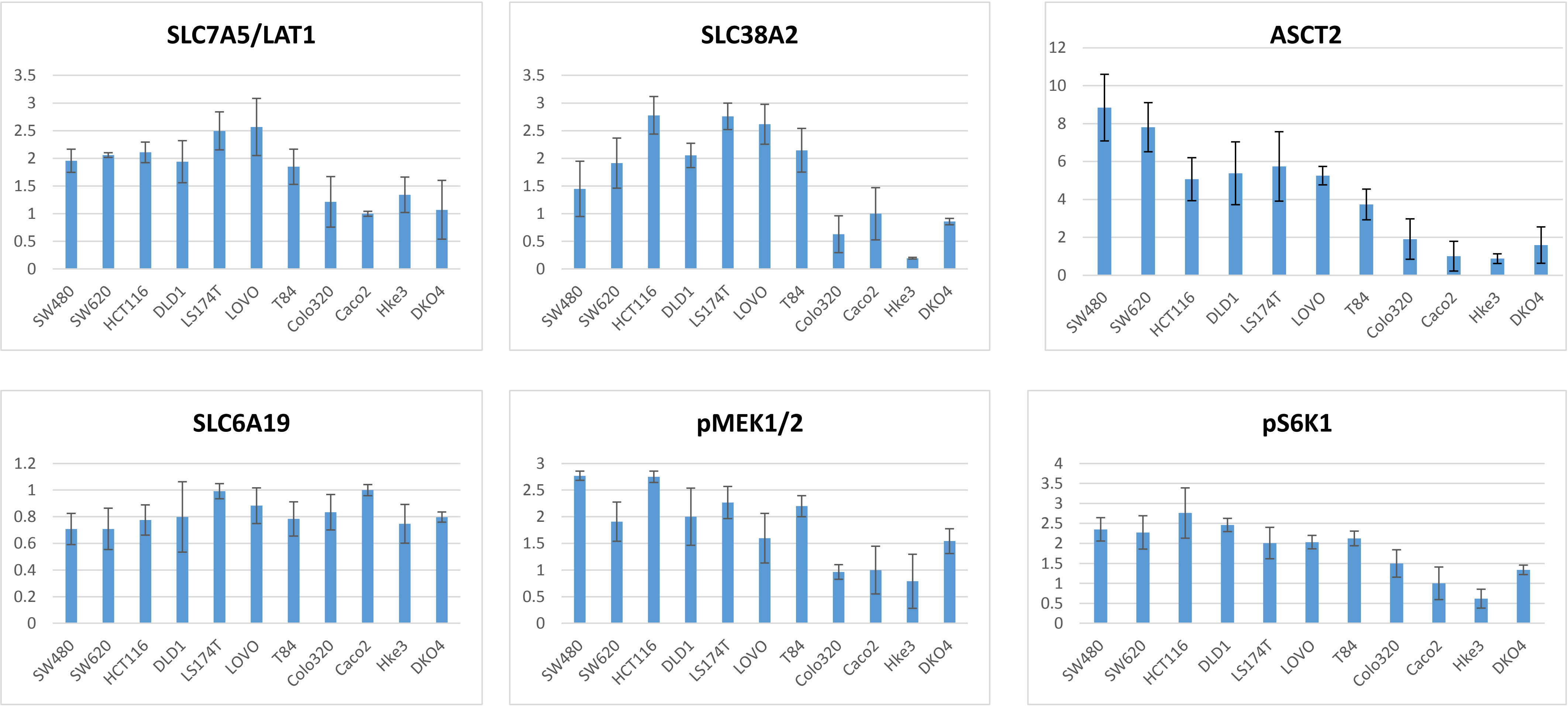

Densitometry for the Figure 1D. Bar graph shows the relative protein expression levels of the targets in different CRC cell lines.

Figure 2A. Western blot quantification

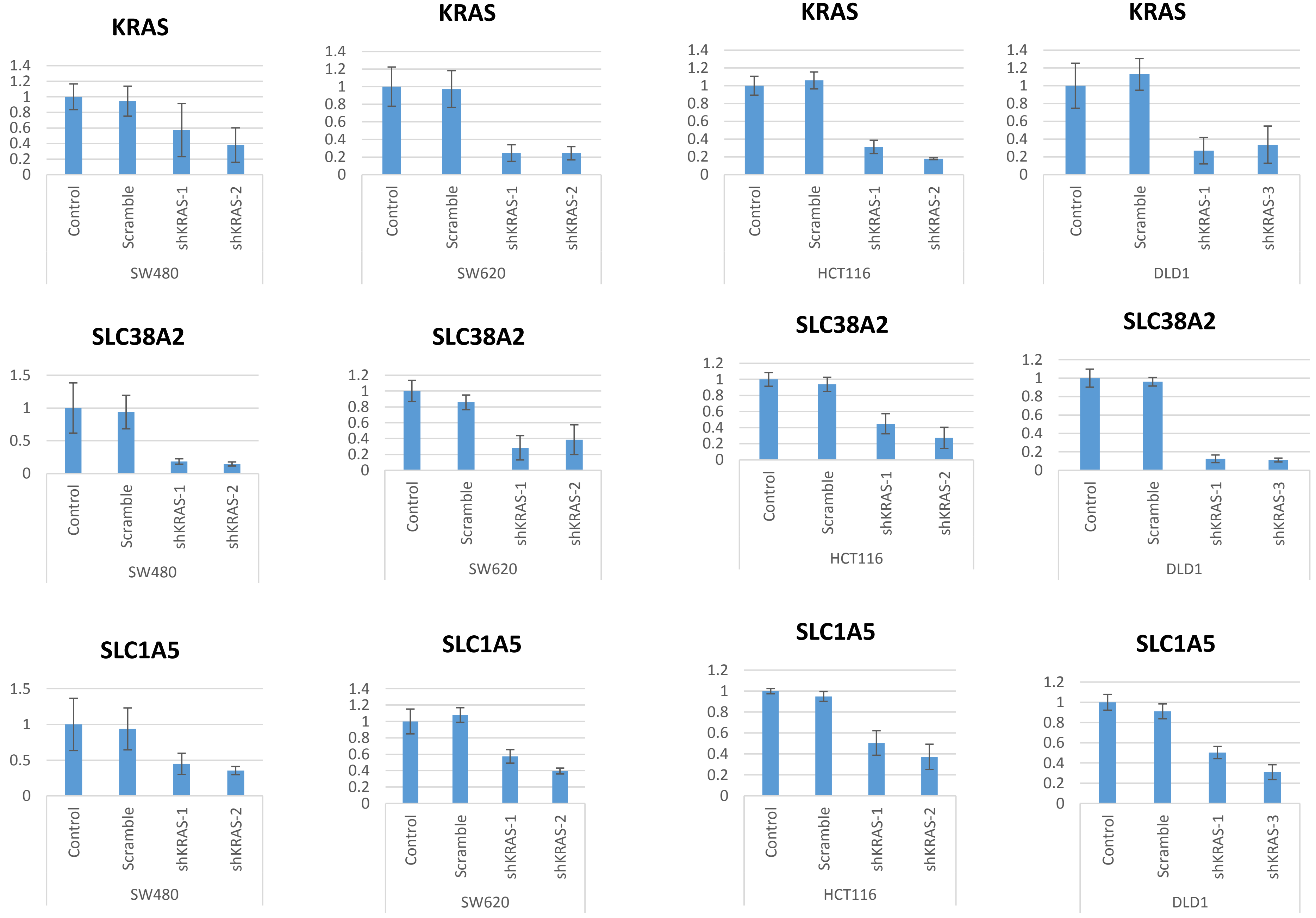

Densitometry for the Figure 2A. Bar graph shows the relative protein expression levels of the targets (KRAS, SLC38A2 and SLC1A5) in shKRAS transfected cells.

Figure 2A. Western blot quantification

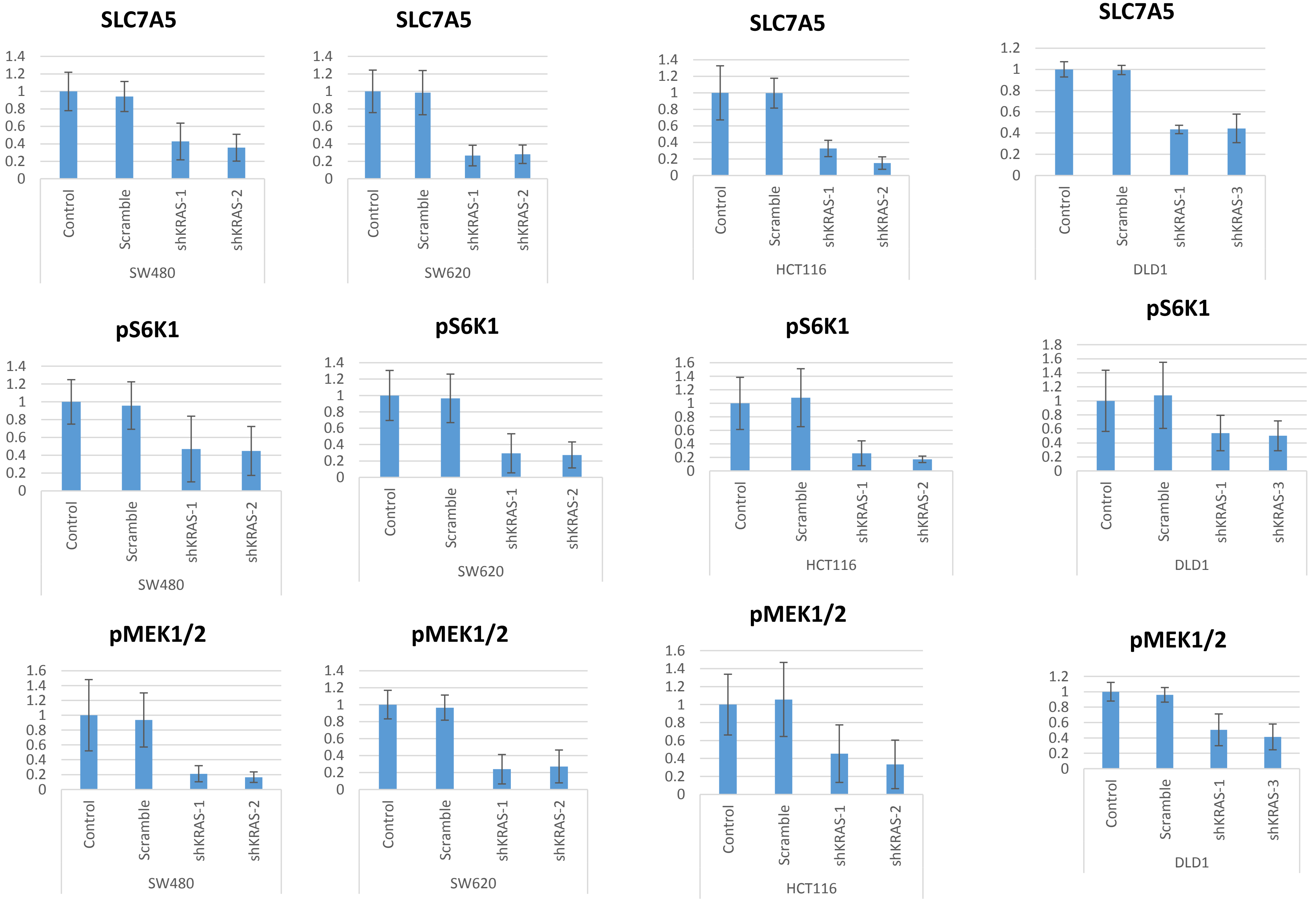

Densitometry for the Figure 2A. Bar graph shows the relative protein expression levels of the targets (SLC7A5, pS6K1and pMEK1/2) in shKRAS transfected cells.

Figure 2A. Western blot quantification

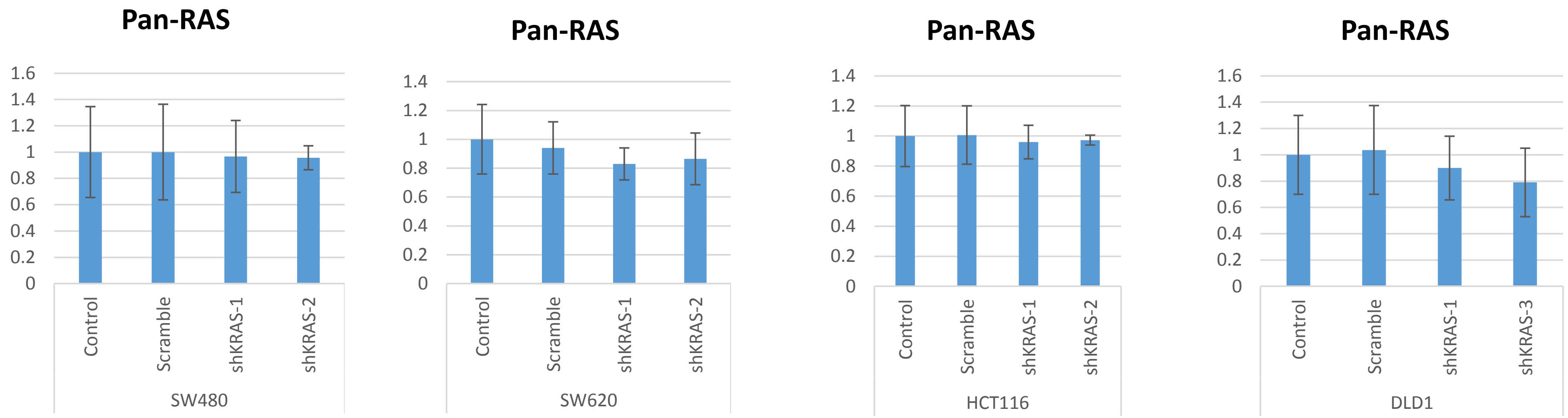

Densitometry for the Figure 2A. Bar graph shows the relative protein expression levels of the targets (pan-Ras) in shKRAS transfected cells.

Fig. 3A. Western blot quantification

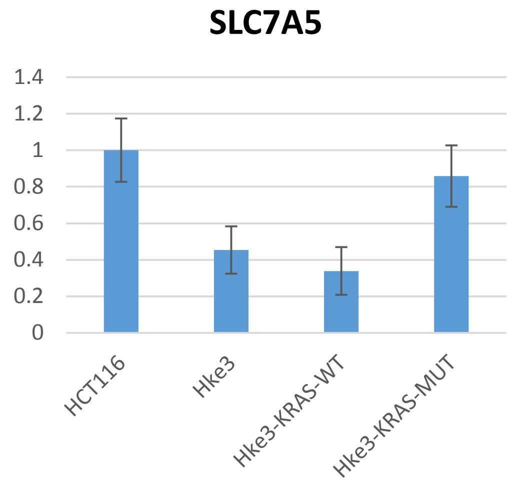

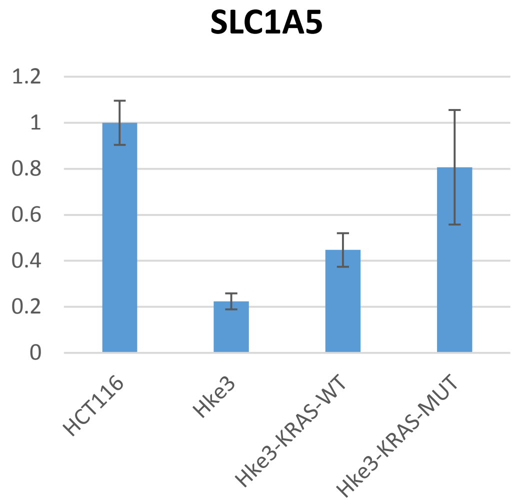

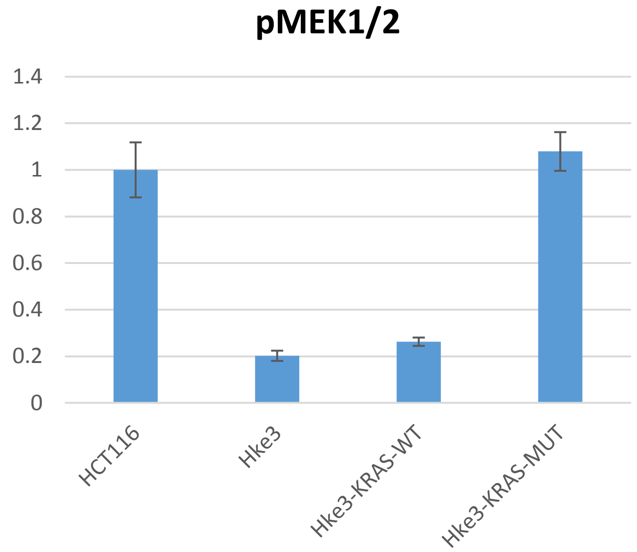

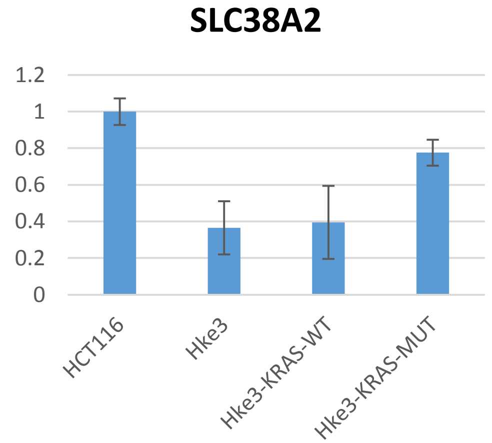

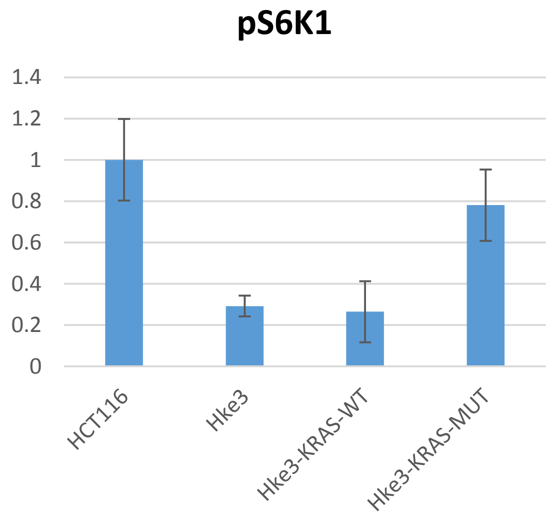

Figure 3A. Bar graph shows the relative protein expression levels of the targets (SLC7A5, SLC1A5, SLC38A2, pS6K1and pMEK1/2) in HCT116 and Hke3 cells.

Supplementary Figure S3A. Western blot quantification

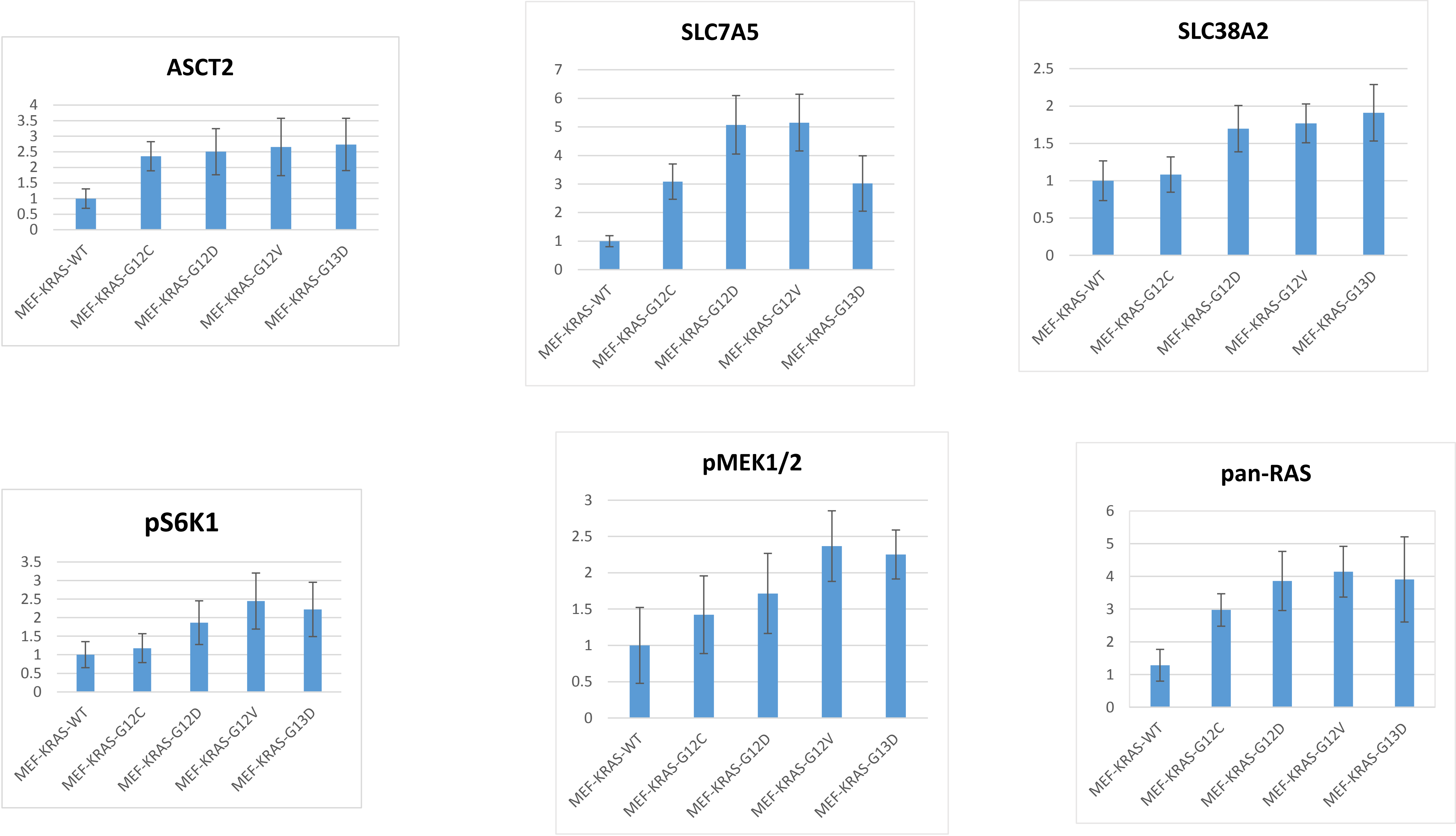

Figure S3A. Bar graph shows the relative protein expression levels of the targets (SLC7A5, SLC1A5, SLC38A2, pS6K1, pan-RAS and pMEK1/2) in MEF-KRAS cells.

Figure 4D. Western blot quantification

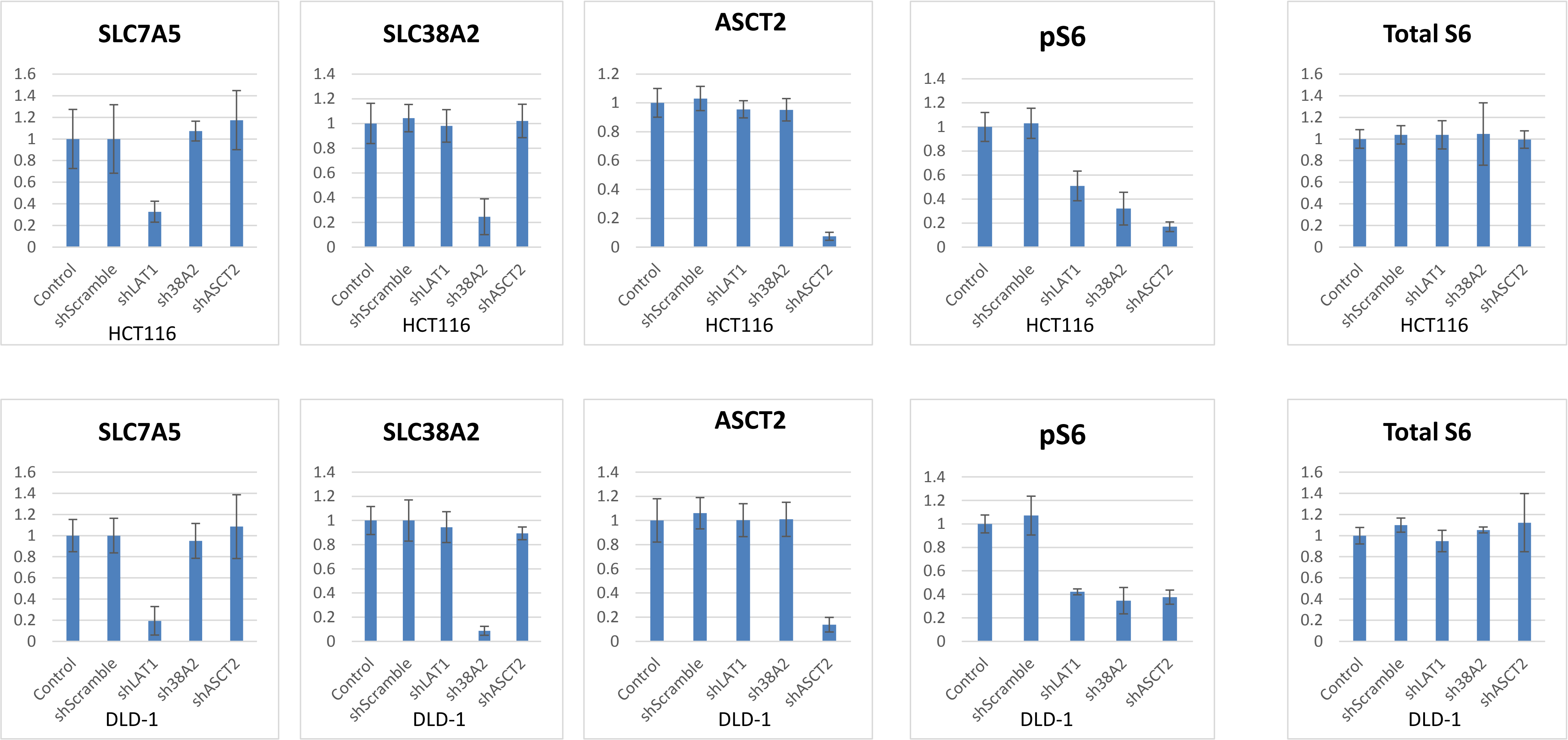

Figure 4D. Bar graph shows the relative protein expression levels of the targets (SLC7A5, SLC1A5, SLC38A2, pS6and total S6) in HCT116 and DLD-1 cells following shRNA transfection against AATs.

Figure 5C and D. Western blot quantification

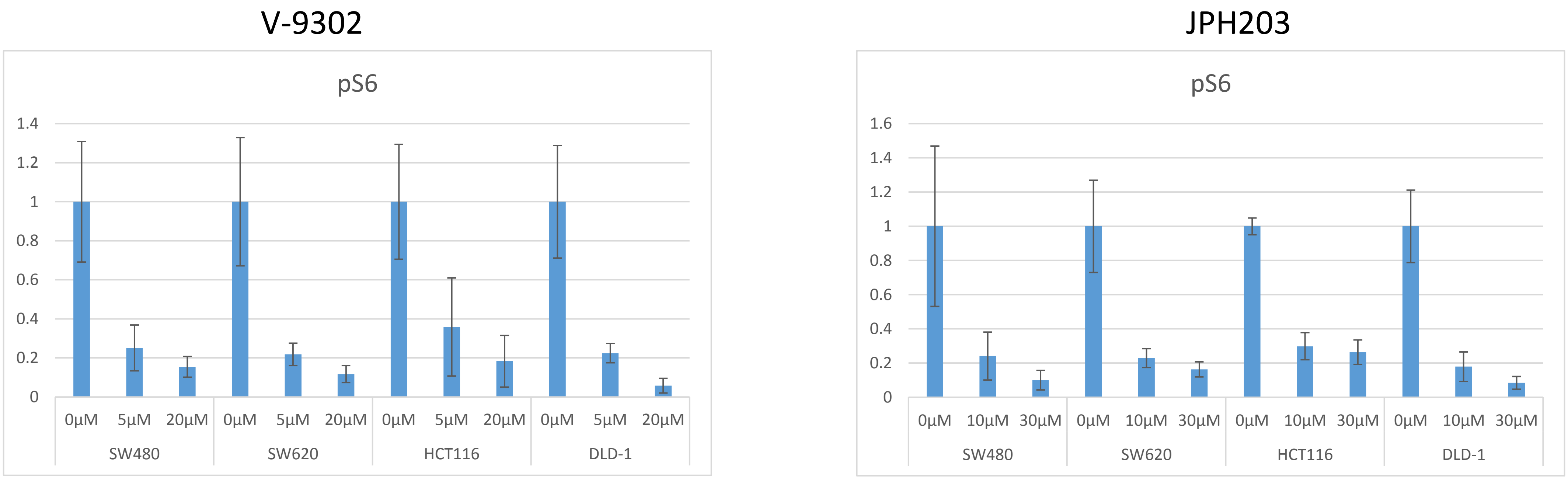

Figure 5C and D. Bar graph shows the relative protein expression levels of the pS6 in different CRC cells following the treatments with the inhibitors of AATs (JPH203 and V-9302).

Supplementary Figure S5.C and D. Western blot quantification

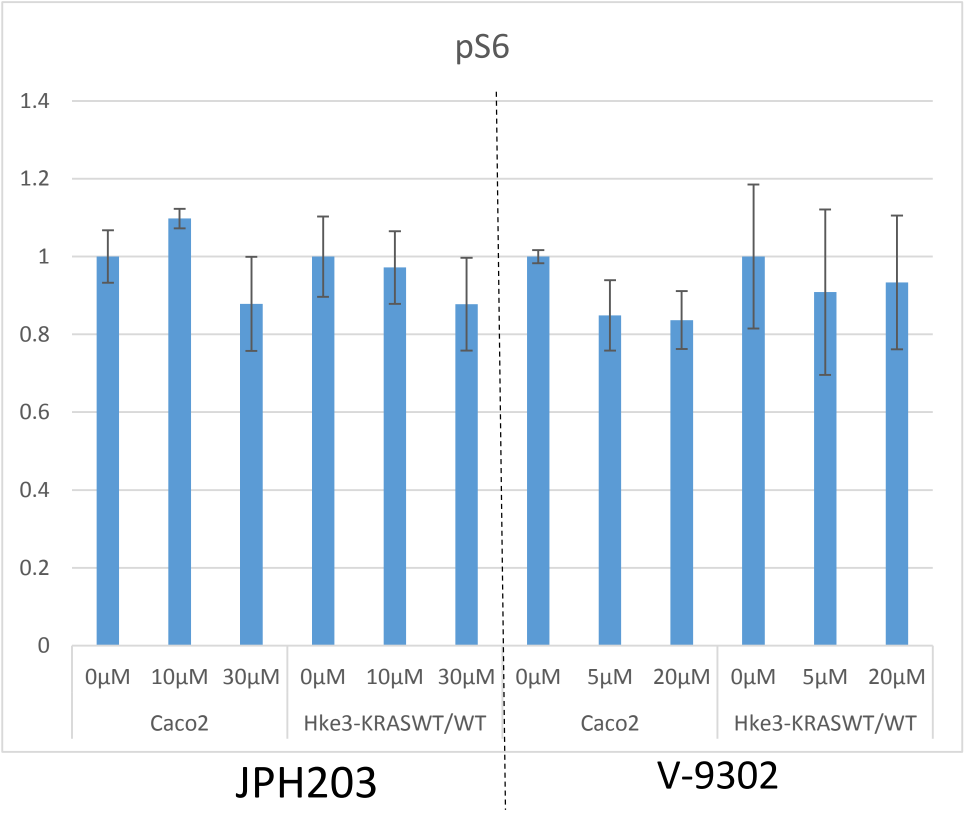

Supplementary Figure 5C and D. Bar graph shows the relative protein expression levels of the pS6 in CRC cells following the treatments with the inhibitors of AATs (JPH203 and V-9302).

Figure 6F. Western blot quantification

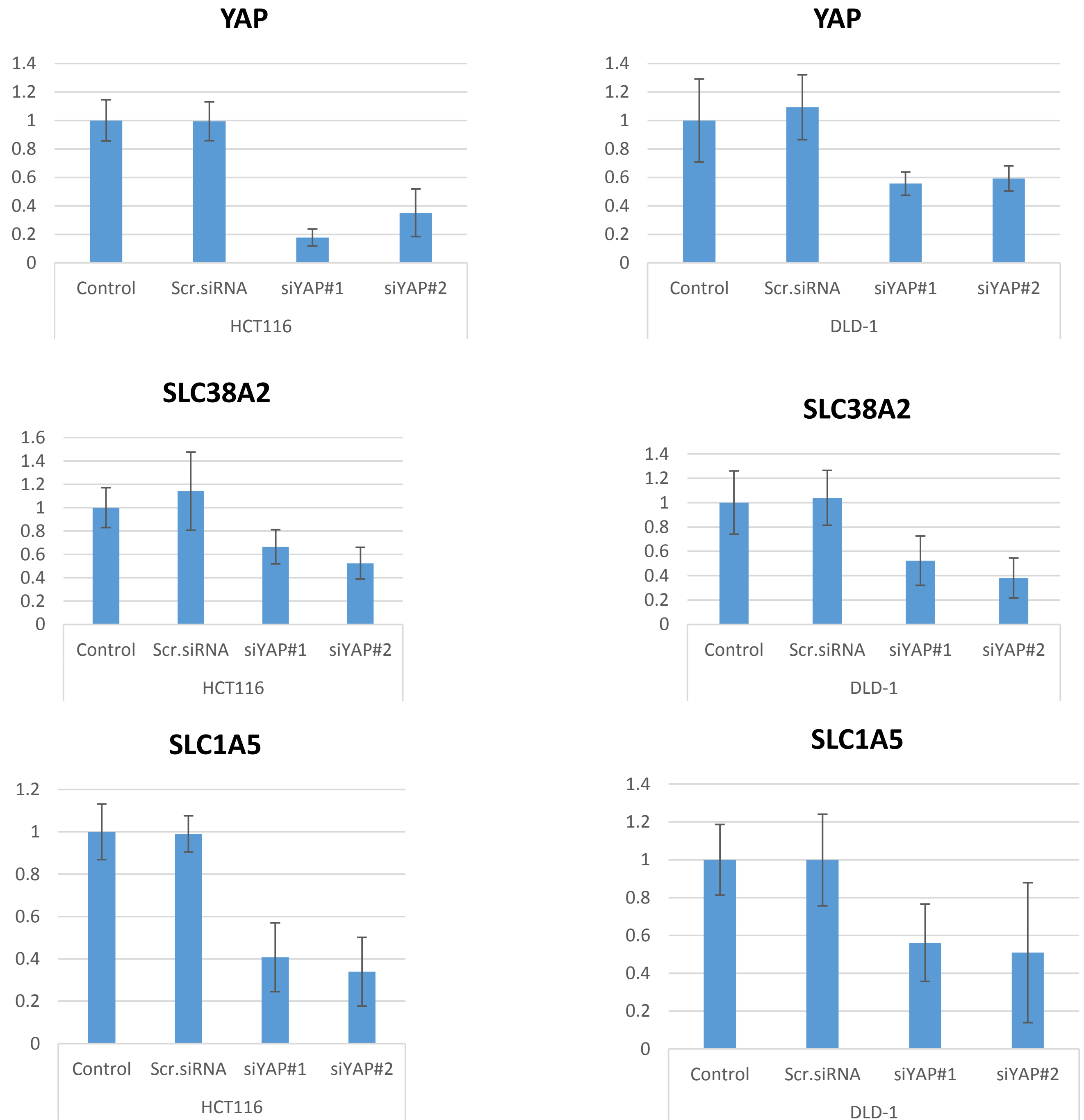

Figure 6. Bar graph shows the relative protein expression levels of the targets (YAP, SLC38A2, SLC1A5) in siYAP transfected cells.

Figure 6F. Western blot quantification

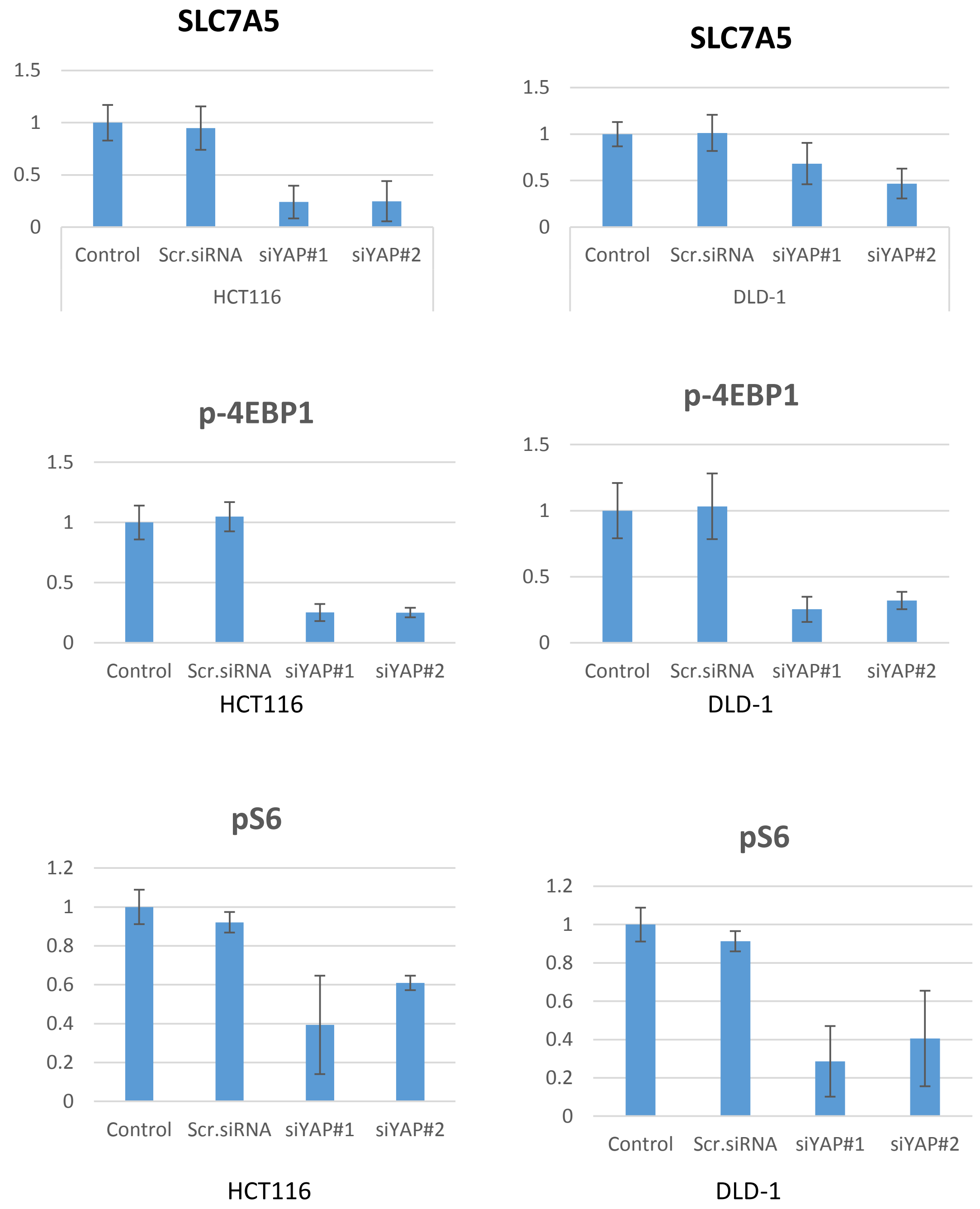

Figure 6. Bar graph shows the relative protein expression levels of the targets (SLC7A5, p-4EBP1, pS6) in siYAP transfected cells.

Figure 6. G. Western blot quantification

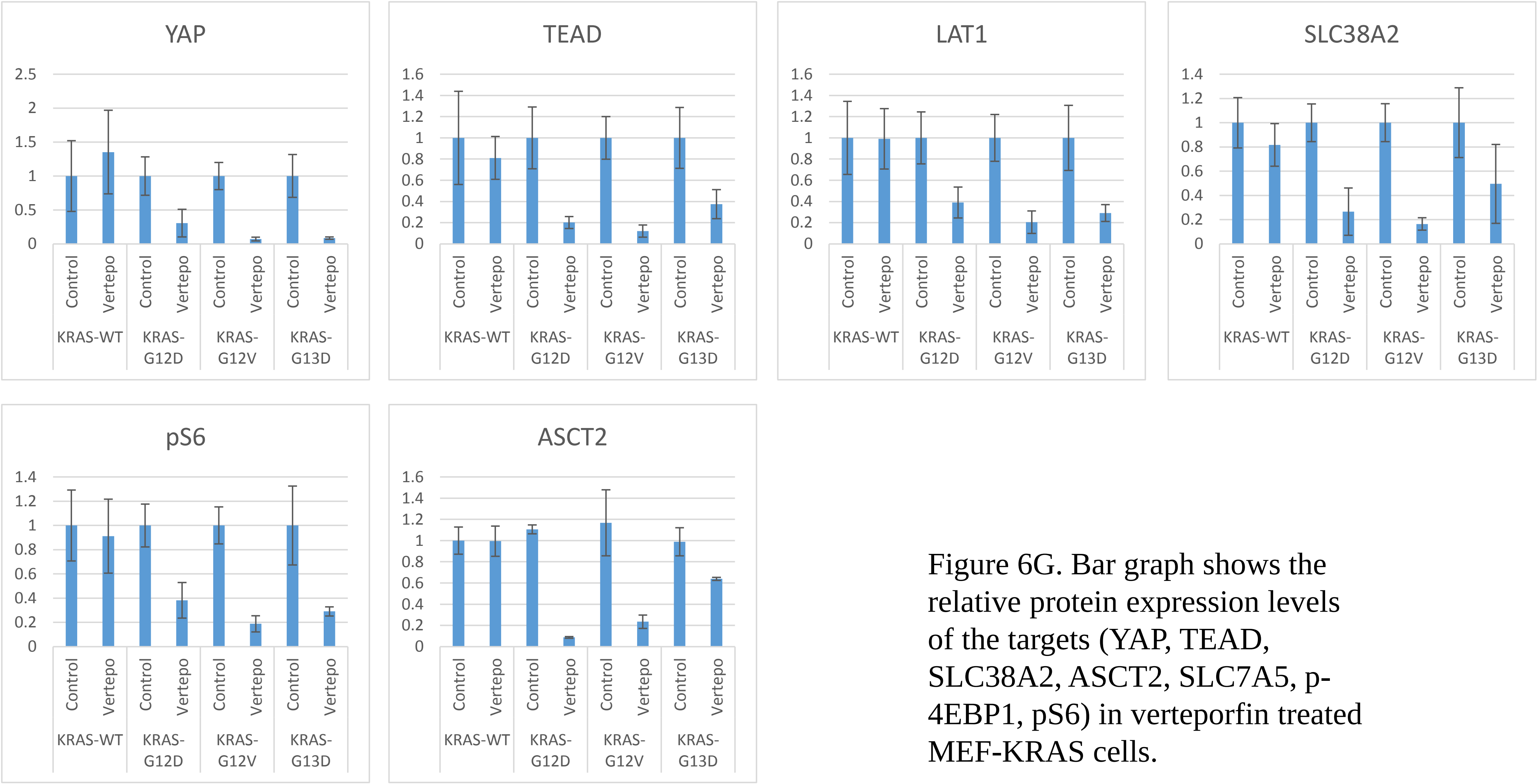

Figure 6G. Bar graph shows the relative protein expression levels of the targets (YAP, TEAD, SLC38A2, ASCT2, SLC7A5, p-4EBP1, pS6) in verteporfin treated MEF-KRAS cells.
